# Enhancing Allicin Purity and Gastrointestinal Bioactivity Profile of Garlic Extracts Through Optimized Supercritical-CO_2_ Extraction and Molecular Distillation Processes

**DOI:** 10.3390/foods15122174

**Published:** 2026-06-16

**Authors:** Hatice Kubra Sasmaz, Pınar Kadiroglu, Turkan Uzlasir, Serkan Selli, Onur Ketenoglu, Hasim Kelebek

**Affiliations:** 1 Department of Food Engineering, Faculty of Engineering, Adana Alparslan Turkes Science and Technology University, 01250 Adana, Türkiye; hkubrasasmaz@hotmail.com (H.K.S.); pkadiroglu@atu.edu.tr (P.K.); 2 Department of Gastronomy and Culinary Arts, Art and Design Faculty, Alanya University, 07400 Alanya, Türkiye; turkan.uzlasir@alanyauniversity.edu.tr; 3 Department of Food Engineering, Faculty of Engineering, Cukurova University, 01330 Adana, Türkiye; sselli@cu.edu.tr; 4 Department of Food Engineering, Faculty of Agriculture, Eskisehir Osmangazi University, 26040 Eskisehir, Türkiye; ketenoglu@gmail.com

**Keywords:** allicin, supercritical CO_2_ extraction, molecular distillation, in vitro digestion

## Abstract

Allicin, the most critical bioactive compound of garlic (*Allium sativum* L.), is of significant industrial importance when extracted at high purity while preserving its structural integrity. In this study, the combined use of supercritical-CO_2_ (SC-CO_2_) extraction and molecular distillation (MD) techniques was investigated to obtain garlic extracts with high allicin content from Gaziantep (Araban) garlic. The SC-CO_2_ extraction process was optimized using Response Surface Methodology (RSM) within a range of 150–300 bar pressure, 50–80% co-solvent concentration and 0.5–3.0 mL/min solvent flow rate. The obtained extracts were characterized by LC-ESI-DAD-MS/MS, and their biological activities were evaluated using a comprehensive in vitro digestion model. Allicin in vitro digestion was performed using models simulating gastrointestinal conditions of young adults (<65 years) and older adults (>65 years), and its bioactive properties were comparatively evaluated. In the antimicrobial analysis, for SC-CO_2_, a strong activity was demonstrated against *Staphylococcus aureus* and *Escherichia coli* in the oral phase of the in vitro digestion model, with inhibition zones of 36.33 mm and 26.50 mm in young samples and 34.67 mm and 25.83 mm in older samples, respectively. Owing to the immediate nucleophilic attack triggered by the subsequent alkaline pH shift and pancreatic enzymatic stress, free allicin underwent total structural degradation, falling below detectable limits within the intestinal chyme. In terms of purification performance, allicin content increased from 45.77% after SC-CO_2_ extraction to 67.10% after molecular distillation. Crucially, due to the immediate nucleophilic attack driven by the subsequent alkaline pH shift and pancreatic enzymatic stress, free allicin underwent complete structural degradation and was rendered strictly undetectable within the intestinal chyme. This approach provides a sustainable and environmentally friendly purification strategy that effectively limits the thermal degradation of allicin. The results present a practical framework for the scalable production of allicin-rich nutraceutical intermediates and functional food ingredients.

## 1. Introduction

Garlic (*Allium sativum* L.) has long been valued not only as an essential culinary ingredient but also as a natural therapeutic agent in traditional medicine [1,2]. Its chemical composition is highly complex, comprising carbohydrates, proteins, amino acids, dietary fibers, and fatty acids, along with various phenolic compounds, essential vitamins (A, B1, B2, niacin, and C), and minerals. Among these constituents, sulfur-containing compounds particularly allicin, alliin, and ajoene stand out as the most characteristic components of garlic [3,4,5,6]. In fact, a wide range of biological activities attributed to garlic, such as antioxidant, antifungal, hypoglycemic, antibacterial, anti-inflammatory, anti-atherosclerotic, and anticancer effects, have been extensively reported in the literature [7,8,9]. These effects are largely associated with organosulfur compounds, with allicin (diallyl thiosulfinate) being recognized as the principal bioactive molecule [10].

Unlike many plant-derived compounds, allicin is not present in intact garlic cloves. Instead, it is rapidly generated following tissue disruption. When garlic is crushed or sliced, the enzyme alliinase, stored in the vacuole, comes into contact with its substrate alliin (S-allyl-L-cysteine sulfoxide), triggering a cascade of reactions that lead to allicin formation [11]. Initially, alliin is converted into allyl sulfenic acid, which then spontaneously condenses to form allicin. Often referred to as the “heart of garlic,” allicin has been shown to exhibit multiple biological activities, including antimicrobial, antioxidant, anticancer, cardioprotective, and antidiabetic effects [12,13]. Nevertheless, its practical use is limited by its high instability. Allicin readily degrades into secondary sulfur compounds such as diallyl disulfide (DADS) and diallyl trisulfide (DATS), even under mild conditions, which makes its extraction and purification particularly challenging [10].

In recent years, there has been increasing interest in developing efficient and environmentally friendly methods for obtaining high-purity natural compounds, especially in the food and pharmaceutical industries. Conventional extraction techniques are often constrained by the use of toxic organic solvents and their associated environmental concerns [14]. Moreover, traditional methods such as steam distillation or solvent extraction are not well suited for thermolabile compounds like allicin, as high temperatures and solvent residues may lead to degradation or contamination [1,15]. In this context, supercritical carbon dioxide (SC-CO_2_) extraction has emerged as a promising alternative. Due to its relatively mild critical conditions (31.1 °C and 7.38 MPa), CO_2_ enables extraction at low temperatures and in oxygen-free environments, thereby reducing thermal and oxidative degradation [15].

Despite these advantages, the allicin purity of SC-CO_2_ extracts remains around 27.79%, which necessitates an additional purification step. Molecular distillation (MD) has therefore attracted attention as an effective technique for this purpose [16]. MD is a specialized form of vacuum distillation that has been used for decades in the pharmaceutical, cosmetic, food, and chemical industries [17]. One of its key strengths is that it allows distillation at significantly reduced temperatures while limiting the exposure time of compounds to heat. As a result, heat-sensitive compounds such as allicin can be separated with minimal degradation [18].

Previous studies have demonstrated that multi-stage MD processes can increase allicin purity from 27.79% up to around 68% [16]. In addition, MD is particularly useful for removing minor impurities and improving the overall quality of thermally unstable compounds while preserving their structural integrity [19,20]. For this reason, the optimization and modeling of MD conditions have become an important focus in recent studies.

For the high-purity extraction of volatile components and bioactive lipophilic hydrophobic segments without degradation, the combination of supercritical carbon dioxide extraction (SC-CO_2_) and MD techniques stands out as an advanced purification strategy. The literature shows that molecular distillation following SC-CO_2_ extraction significantly improves the fluidity and purity of the product by removing high molecular weight waxes, heavy components, and pigments carried by co-extraction in crude extracts. In a pilot-scale separation of *Artemisia argyi* essential oils, the integration of SC-CO_2_ and MD has been reported to completely eliminate the risk of thermal decomposition and hydrolysis compared to conventional hydrodistillation methods, and to provide high-purity fractionation while preserving the natural major composition of the vegetable oil [21]. Similarly, it has been found that using a short-path molecular distillation system in the purification of heat-sensitive microalgae (*Nannochloropsis gaditana*) lipids approximately doubles the unsaturated fatty acid (particularly EPA) concentration (from 29.88% to 58.63%) and overcomes bottleneck problems encountered in conventional methods [22].

To date, contemporary literature on allicin recovery frequently suffers from a critical fragmentation: studies either focus on crude extraction without downstream purification or rely on conventional thermal separation methods that inevitably trigger the degradation of this highly thermolabile thiosulfinate core [16,22,23,24]. Therefore, the aim of this study was to develop and optimize an integrated two-stage green processing platform combining SC-CO_2_ extraction and short-path molecular distillation for the production of high-purity allicin-rich fractions from Gaziantep garlic using RSM. In addition, the physicochemical properties, antioxidant activity, DNA-protective capacity, antimicrobial activity, and gastrointestinal bioaccessibility of the purified fractions were comprehensively evaluated using age-specific in vitro digestion models based on the standardized INFOGEST protocol.

## 2. Materials and Methods

### 2.1. Material

The garlic samples used in this study were obtained from Gaziantep garlic cultivated in the Araban Plain (37°24′ N; 37°57′ E), located in the Araban district of Gaziantep Province, Türkiye. The Araban Plain is an important agricultural production area and a significant source of regional income. The samples consisted of dried garlic from the June 2023 harvest season, which was purchased from local markets after the completion of post-harvest drying by the producers. The initial moisture content of the garlic was determined to be 71.06%. When garlic is stored as whole bulbs, its natural protective outer layers limit moisture loss and microbial growth. Therefore, the garlic samples used in this study were stored as whole bulbs in a dark, dry environment with adequate airflow at 15 °C.

### 2.2. Experimental Design for Supercritical CO_2_ Extraction (SC-CO_2_)

An optimization study was conducted to produce garlic with a high allicin content under optimal conditions. A limited number of articles found in the literature were reviewed, and the lower and upper limits of the pressure, co-solvent concentration, and flow rate studied were used as independent variable levels in the extraction phase. In this proposed study, the experimental design response surface method was applied using the rotatable Box–Behnken design with 3 factors, implemented with the Design Expert (Stat-Ease, Design-Expert software, version 13) program. The independent variables were defined as co-solvent concentration (50–80%, *v*/*v*), pressure (150–300 bar), and flow rate (0.5–3 mL/min) (Table 1). Garlic samples were ground using a Waring Commercial blender (Waring Commercial, Stamford, CT, USA). As a result of the grinding process, the samples were reduced to a particle size of approximately 1–2 mm. Fresh garlic samples (100 g) were peeled and homogenized prior to extraction. The extraction temperature was set at 45 °C and the extraction time was 120 min. Extractions were performed using the Superex F-500 Supercritical Extraction System (PARD Engineering, Konya, Türkiye) according to the experimental design given in Table 1. The prepared garlic samples were loaded into the extraction vessel and processed under the specified conditions defined by the design. To systematically evaluate and mathematically model the extraction efficiency of target organosulfur formulations from *Allium sativum* L., a three-factor, three-level rotatable Box–Behnken design (BBD) was implemented. The boundary conditions for the independent variables—namely, extraction pressure (X_1_: 150–300 bar), co-solvent concentration (X_2_: 50–80%, *v*/*v*), and flow rate (X_3_: 0.5–3.0 mL/min)—were rigorously established based on preliminary single-factor exploratory assays and screening of thermodynamic limitations associated with allicin stability. An ethanol–water mixture was used as a co-solvent in the extraction process. Co-solvent concentration was defined as the volumetric concentration of ethanol within the binary ethanol–water co-solvent mixture (50–80%, *v*/*v*), rather than the total modifier-to-CO_2_ ratio. After extraction, samples were stored at −20 °C to preserve allicin stability until analysis. To minimize allicin degradation, all samples were stored at −20 °C in airtight amber vials to prevent exposure to light and oxygen. The time between extraction and analysis was kept as short as possible. Repeated freeze-thaw cycles were avoided. Sample preparation prior to analysis was carried out on ice to reduce allicin degradation. The responses evaluated included allicin content, total phenolic content (TPC), antioxidant capacity (DPPH and ABTS), and total sulfur compounds (TSC). These responses were analyzed using the software, and optimization was achieved by maximizing allicin, TSC, TPC, and antioxidant activity across 17 experimental runs. A mathematical model was developed using multiple regression analysis, and the significance of model terms was assessed by ANOVA. Model adequacy was confirmed by a non-significant lack-of-fit and a significant F-test (*p* < 0.05). The optimal extraction conditions yielding the highest allicin content were determined from the experimental design. To validate the model, the extraction was repeated three times under optimal conditions, and the corresponding response values were verified.

The extract obtained under optimal extraction conditions, where the highest level of allicin is achieved, was subjected to molecular distillation to obtain allicin of higher purity.

### 2.3. Experimental Design for Molecular Distillation (MD)

Allicin was purified from extracts with high allicin content obtained from garlic under optimal conditions using SC-CO_2_ support via molecular distillation. Short-path MD was conducted using a KDL1 laboratory-scale short-path evaporator with an evaporation surface area of 0.01 m^2^ (UiC GmbH, Alzenau, Germany) under moderate vacuum (70–250 mbar). This operating pressure range was deliberately selected to balance the mechanical limitations of the system with the evaporation kinetics of the garlic organosulfur fractions, preventing the thermal collapse of allicin while maintaining scalable throughput. The operating characteristics and conditions were given in Table 1. The independent variables in the experiments were: wiper speed (rpm), feed rate (mL/min), pressure (mbar), evaporator unit temperature (°C), and condenser temperature (°C). A total of 45 experiments were conducted, and the purity of allicin obtained in each experiment was determined as the response variable. To systematically model the downstream enrichment of the organosulfur fractions, Response Surface Methodology (RSM) was deployed using a five-factor Box–Behnken design. Within this mathematical design framework, allicin purity (%) was defined as the sole primary response variable (Y) to execute the quadratic polynomial optimization and surface modeling. The experimental design created using the RSM was implemented using Design-Expert software (version 13.0, Stat-Ease Inc., Minneapolis, MN, USA).

### 2.4. Determination of Allicin and Total Sulphur Compounds

Quantitative analysis of allicin and total organosulfur compounds was performed using an Agilent 1260 LC system equipped with a diode array detector and coupled to an Agilent 6460 triple quadrupole mass spectrometer (Agilent Technologies, Santa Clara, CA, USA). Chromatographic separation was achieved on a reversed-phase C18 column (Phenomenex Luna, 250 × 4.6 mm, 5 μm; Torrance, CA, USA). The mobile phase consisted of two solvents: Solvent A, water containing formic acid (99:1, *v*/*v*) (Sigma-Aldrich, Merck, St. Louis, MO, USA), and Solvent B, a mixture of Solvent A and acetonitrile (60:40, *v*/*v*) (Sigma-Aldrich, Merck, St. Louis, MO, USA). The elution program was as follows: 0–5 min, 5% B (isocratic); 5–10 min, 5–15% B; 10–25 min, 15–20% B; 25–35 min, 20–25% B; 35–50 min, 25–40% B; and 50–65 min, 40–55% B. The gradient was then held at 55% B for 5 min, followed by 55–70% B (25 min), 70–100% B (1 min), and 100% B for 9 min. At the end of the run, the mobile phase was returned to 5% B within 1 min for column washing and re-equilibration. The flow rate was set to 0.5 mL/min, and the column temperature was maintained at 25 °C. UV-visible spectra between 200 and 800 nm were recorded for peak monitoring. Compound identification was achieved by comparing retention times and UV spectral characteristics with analytical standards. For quantitative verification, samples were additionally analyzed by LC-MS/MS [25]. The electrospray ionization (ESI) source parameters were systematically optimized by setting the nebulizer pressure to 45 psi, a drying gas flow of 15 L/min, and a source temperature of 400 °C. For each target analyte, capillary and cone voltages were individually adjusted, and collision energies were fine-tuned to achieve optimal signal response and analytical sensitivity. The quantitative analysis of sulfur-containing metabolites including alliin, (+)-S-allyl-L-cysteine (SAC), and γ-L-glutamyl-phenylalanine (γGPA) were obtained from Sigma-Aldrich (Sigma-Aldrich, Merck, St. Louis, MO, USA), while γ-L-glutamyl-S-allyl-L-cysteine (GSAC) and allicin were obtained from Santa Cruz Biotechnology (Dallas, TX, USA). γ-L-glutamyl-S-(trans-1-propenyl)-L-cysteine (GSPC) was purchased from MedChemExpress (Monmouth Junction, NJ, USA). Multiple reaction monitoring (MRM) transitions were optimized for each compound as follows: alliin (*m*/*z* 178.1 → 88.1), GSAC (*m*/*z* 291.2 → 162.2 and 144.8), GSPC (*m*/*z* 291.2 → 201.1), γGPA (*m*/*z* 295.3 → 178.0 and 88.0), SAC (*m*/*z* 162.1 → 145.1 and 73.1), and allicin (*m*/*z* 163.2 → 73.2 and 41.1). Method performance was assessed through linearity (R^2^), limits of detection (LOD), limits of quantification (LOQ), and repeatability tests. The LOD and LOQ were determined to be in the ranges of 0.3–28.8 ng/mL and 1.0–87.2 ng/mL, respectively. Excellent linearity was obtained for all calibration curves, with correlation coefficients exceeding 0.99, demonstrating strong sensitivity, precision, and reliability of the method. The purity of allicin was determined using the following equation:

Allicin purity (%) = (Allicin amount/Total sulphur compounds) × 100

### 2.5. Determination of Antioxidant Capacity and Total Phenolic Content

The antioxidant capacity of the samples was evaluated using the DPPH (Sigma-Aldrich, Merck, St. Louis, MO, USA) and ABTS (Sigma-Aldrich, Merck, St. Louis, MO, USA) radical scavenging assays. In the DPPH (2,2-diphenyl-1-picrylhydrazyl) assay, the radical reduction potential of the extracts was assessed by monitoring the change in absorbance in a methanolic medium at 515 nm using a UV-Vis spectrophotometer (Agilent Cary 60, Agilent Technologies, Santa Clara, CA, USA) [6,26].

The ABTS radical scavenging activity was determined following the procedure described by Sasmaz et al. [25]. The results were calculated using a Trolox (Sigma-Aldrich, Merck, St. Louis, MO, USA) calibration curve prepared in the concentration range of 10–100 μmol/L, and expressed as mmol/L Trolox equivalents.

The CUPRAC assay was carried out according to the method described by Apak et al. [27]. In brief, the reaction mixture contained 1.0 × 10^−2^ M copper(II) chloride (CuCl_2_·2H_2_O) (Sigma-Aldrich, Merck, St. Louis, MO, USA), 1 M ammonium acetate (Sigma-Aldrich, Merck, St. Louis, MO, USA) buffer adjusted to pH 7.0, and 7.5 × 10^−3^ M neocuproine (2,9-dimethyl-1,10-phenanthroline) (Sigma-Aldrich, Merck, St. Louis, MO, USA) solutions. A 1.0 × 10^−3^ M Trolox solution was used for the calibration curve. First, 1 mL of Cu(II), 1 mL of neocuproine, and 1 mL of buffer were added sequentially into test tubes, after which 0.5 mL of sample extract or 0.3 mL of liquid sample was introduced. The total reaction volume was brought to 4.1 mL with distilled water. The mixtures were vortexed thoroughly and incubated for 30 min at room temperature in the dark. Absorbance readings were taken at 450 nm against a reagent blank, and the antioxidant capacity was calculated as mmol Trolox equivalents per gram of sample (mmol TE/g) using the Trolox standard calibration curve.

The FRAP (Ferric Reducing Antioxidant Power) assay was performed based on the procedure of Benzie and Strain [28]. This method is based on the reduction in ferric ions (Fe^3+^) to ferrous ions (Fe^2+^) in the presence of antioxidants, resulting in the formation of a blue Fe^2+^TPTZ complex. The absorbance of the resulting solution was measured at 593 nm, and antioxidant activity was expressed as mmol Fe^2+^ equivalents per gram of sample.

Total phenolic content (TPC) was determined using the Folin–Ciocalteu method as described by Sasmaz et al. [25]. In this assay, 200 μL of either sample or standard solution was mixed with Folin–Ciocalteu reagent (Sigma-Aldrich, Merck, St. Louis, MO, USA) diluted at a ratio of 1:10. After allowing the reaction to stand for 5 min, 1.5 mL of 6% sodium carbonate (Sigma-Aldrich, Merck, St. Louis, MO, USA) solution was added. The mixture was then incubated for 90 min at room temperature in the dark. Finally, absorbance was recorded at 765 nm using a UV-Vis spectrophotometer.

### 2.6. Determination of Antibacterial Activity and Minimum Inhibitory Concentration (MIC)

The antimicrobial profiles of the raw fluid extracts and purified allicin fractions were rigorously screened using a sequential combination of the agar well diffusion assay and the broth microdilution technique, systematically aligned with the clinical guidelines of the Clinical and Laboratory Standards Institute (CLSI, M02-A13 and M07-A11 protocols).

Inoculum Preparation and Incubation Conditions: Prior to testing, the bacterial strains (*Staphylococcus aureus* ATCC 29213, *Pseudomonas aeruginosa* ATCC 27853, *Escherichia coli* ATCC 25922, *Bacillus subtilis* ATCC 11774, and *Klebsiella pneumoniae* ATCC 13883) were obtained from Microbiologics (St. Cloud, MN, USA) and were refreshed on Mueller-Hinton Broth (MHB) at 37 °C for 18–24 h. Standardized microbial suspensions were prepared in sterile saline (0.85% NaCl) and adjusted photometrically to match the 0.5 McFarland turbidity standard, yielding an approximate density of 1.5 × 10^8^ CFU/mL for bacteria.

Agar Well Diffusion Assay: The standardized inocula were uniformly seeded onto Mueller–Hinton Agar (MHA) plates using sterile cotton swabs. Wells (6.0 mm in diameter) were aseptically prepared using a sterile borer. Subsequently, 50 µL of each garlic fraction dissolved in the optimized solvent system was added to the wells. To establish strict empirical accountability, solvent controls consisting of the exact hydro-ethanolic extraction baseline (without allicin) were simultaneously introduced to confirm the absence of intrinsic vehicle toxicity. Commercial antibiotics (Gentamicin and Nystatin at a locked concentration of 10 µg/mL) were obtained from Bioanalyse (Ankara, Turkey) and served as the standard positive controls. The plates were incubated in an inverted position at 37 °C for 24 h, after which the clear zones of inhibition were quantified using a digital vernier caliper.

Broth Microdilution for MIC Determination: To determine the precise Minimum Inhibitory Concentration (MIC) values within the CLSI-type framework, automated broth microdilution was executed in sterile 96-well flat-bottom microplates [29]. The samples were prepared as two-fold serial dilutions within the concentration range of 30–0.05 mg/mL, loaded into the wells, and incubated at 35 °C for 18–24 h. All assays were performed in duplicate, and the diameters of the inhibition zones were measured three times after incubation. The extracts were added to Muller–Hinton broth containing test microorganisms’ solutions, adjusted to a 0.5 McFarland value [5]. The MIC was formally defined as the lowest absolute concentration of the optimized extract that visually inhibited 100% of microbial proliferation, subsequently validated spectrophotometrically at 600 nm.

### 2.7. Determination of Antifungal Activity

The antifungal activity of the allicin-enriched extract and purified allicin was evaluated by the disk diffusion method based on the protocol described by Alastruey-Izquierdo, and Cuenca-Estrella [30]. The fungal strains used in the study were *Aspergillus fumigatus*, *Penicillium chrysogenum*, *Cladosporium cladosporioides*, and *Alternaria* species and these fungal strains were obtained from Microbiologics (St. Cloud, MN, USA). The molds were cultivated on Malt Extract Agar at 30 °C for 5–7 days to obtain fresh cultures. Colonies were suspended in peptone water and adjusted to McFarland standard 0.5 to achieve a final inoculum density of approximately 10^6^–10^7^ CFU/mL. For inoculation, 0.1 mL of each fungal suspension was spread onto the surface of modified Mueller-Hinton Agar (MHA) supplemented with 2% glucose and 0.5 mg/L methylene blue using a Drigalski spatula. Following inoculation, plates were left at room temperature for 10 min to allow uniform distribution. The prepared samples were loaded onto sterile paper disks at defined concentrations, dried at room temperature, and placed on the agar surface, ensuring that no contact occurred between the disks. Plates were incubated at 30 °C for 4–7 days, after which inhibition zone diameters were measured in millimeters using a digital caliper under sufficient natural light. All analyses were performed in triplicate to ensure data reliability.

### 2.8. Determination of DNA Damage Protective Capacity

The DNA protective potential of the allicin-rich extract and pure allicin was evaluated by agarose gel electrophoresis based on the photolysis of supercoiled pBR322 plasmid DNA with H_2_O_2_ under UV irradiation. Reaction mixtures containing 3 μL of pBR322 plasmid DNA (Thermo Scientific, Waltham, MA, USA), 5 μL of sample, and 1 μL of 30% H_2_O_2_ (Sigma-Aldrich, Merck, St. Louis, MO, USA) were prepared in microtubes. Untreated DNA served as the control, while reactions containing DNA and H_2_O_2_ without the sample were used as negative controls. Tubes were irradiated under a UV transilluminator (320 nm) for 5 min at room temperature. After irradiation, 2 μL of loading dye was added, and the samples were analyzed by electrophoresis on 0.8% agarose gels prepared in TAE buffer (pH 8) containing ethidium bromide and subsequently visualized. The analysis was performed in triplicate [31].

### 2.9. Determination of Bioaccessibility by In Vitro Gastrointestinal Digestion

In this study, two distinct static in vitro gastrointestinal digestion models were applied to represent different age groups, considering the age-dependent changes in digestive physiology. The first model represents younger adults <65 years of age, while the second corresponds to older adults >65 years. Both models were designed and conducted according to the INFOGEST consortium protocols with age-specific modifications [32,33]. All chemicals used in the in vitro digestion assay were obtained from Sigma-Aldrich (Sigma-Aldrich, Merck, St. Louis, MO, USA). Younger adults (<65 years) model: A 5 g sample was mixed with simulated salivary fluid (SSF, 1:1 *w*/*w*) containing α-amylase (75 U/mL) and CaCl_2_ and incubated at 37 °C for 2 min. In the gastric phase, the oral bolus was combined with simulated gastric fluid containing pepsin (2000 U/mL), adjusted to pH 3.0, and incubated at 37 °C for 2 h. In the intestinal phase, the gastric content was mixed with simulated intestinal fluid containing pancreatin (100 U/mL) and bile salts (10 mM), adjusted to pH 7.0, and incubated at 37 °C for 2 h. In the older adult (>65 years) model, the oral phase was left unchanged; however, physiological modifications were applied to the gastric and intestinal phases. In the gastric phase of the older adult model, pepsin activity was reduced to 1200 U/mL, pH was adjusted to 3.7, and incubation time was extended to 3 h. In the intestinal phase, pancreatin (80 U/mL) and bile salts (6.7 mM) were used, while other conditions remained unchanged. A blank control was included for background correction, and all experiments were performed in triplicate.

### 2.10. Statistical Analysis

The optimization process was performed using Response Surface Methodology (RSM) combined with a Box–Behnken Design to determine the optimal levels of the variables and achieve the desired response. Analysis of variance (ANOVA) was employed to evaluate the adequacy of both the experimental data and the proposed model. The significance of model terms was assessed using F-values, and variables that were not statistically significant (*p* > 0.05) were removed from the model. Following their elimination, the regression coefficients were recalculated to improve model accuracy. For data streams generated from the non-RSM operations, including physicochemical properties (pH, L*, a*, b* color parameters), TPC, multi-mechanism antioxidant capacity (DPPH, ABTS, CUPRAC, FRAP), phase-dependent in vitro gastrointestinal bioaccessibility segments, quantitative DNA densitometric tracking, and broad-spectrum antibacterial/antifungal zone susceptibility profiles, all assays were executed across exactly three independent replicates (*n* = 3) with technical triplicates. The significance of differences among the independent variables and digestion phases was assessed using One-Way Analysis of Variance (ANOVA). For all analytical, biological, and molecular screenings, the threshold for statistical significance was strictly locked at a confidence level of 95% (*p* < 0.05). All statistical computations were performed using SPSS Statistics (v26.0; IBM Corp., Armonk, NY, USA).

## 3. Results

### 3.1. Integrated Optimization of SC-CO_2_ Extraction and MD for High-Allicin Garlic Extract

#### 3.1.1. Optimization of SC-CO_2_ Extraction

**Figure 1:**
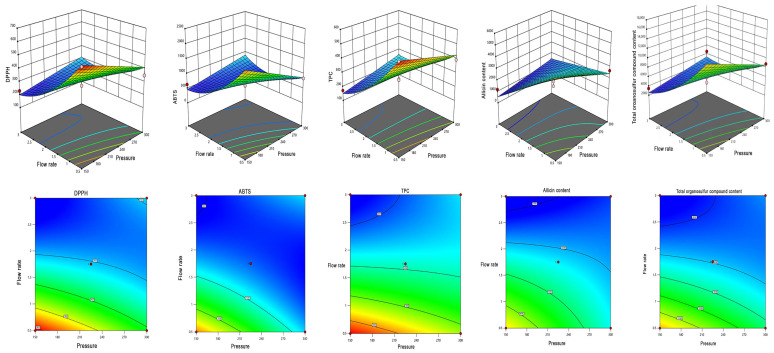
Contour and response surface plots showing the interaction effects of SC-CO_2_ extraction variables under optimized experimental design.

In the present study, SC-CO_2_ extraction was employed to obtain bioactive compounds from garlic. Extractions were conducted using 100 g of fresh garlic according to the experimental design presented in Table 1. Accordingly, the model’s suitability was ensured by the lack of fit value-derived error being insignificant and the variation resulting from the regression being significant at the 95% confidence interval. In determining the optimum extraction conditions, the desirability function method was also utilized, along with response surface curves [34]. The experimental design for the SC-CO_2_ extraction method is presented in Table 1, and the results obtained from the optimization study are presented in Table S1. Contour and response surface plots illustrating the interaction effects of SC-CO_2_ extraction variables under an optimized experimental design are shown in Figure 1.

The DPPH radical scavenging capacity ranged from 229.95 to 605.28 µmol Trolox/L (Table S1). The highest DPPH value was determined in the extract obtained at a pressure of 150 bar, a co-solvent concentration of 65%, and a flow rate of 0.5 mL/min. The ANOVA results for the reduced Box–Behnken design model are presented in Table S2. The lack-of-fit term was not significant (*p* = 0.1783), indicating that the model adequately fits the experimental data. The overall model was highly significant (*p* < 0.0001), confirming that the selected factors collectively have a strong effect on DPPH activity. Among the model terms, flow rate (C) (*p* < 0.0001), the pressure × flow rate interaction (AC) (*p* = 0.0044), and the quadratic term of flow rate (C^2^) (*p* = 0.0037) were statistically significant. In contrast, pressure (A) did not show a significant effect on the response (*p* = 0.1688). The adequacy of the reduced model was further evaluated using various statistical indicators. The model exhibited a high coefficient of determination (R^2^ = 0.8863), explaining 88.63% of the variation in DPPH values. The adjusted R^2^ value (0.8484) was in good agreement with the R^2^ value, confirming the adequacy of the selected model terms. The predicted R^2^ value (0.6787) indicated that the model has acceptable predictive ability for new observations. The relatively low coefficient of variation (CV = 13.08%) demonstrated good precision and reliability of the experimental results. Furthermore, the adequate precision value of 17.0706, which is well above the desirable threshold of 4, confirmed an adequate signal-to-noise ratio and indicated that the response can be predicted reliably. The standard deviation and PRESS values were calculated as 44.80 and 68,070.09, respectively, further supporting the predictive performance of the developed model (Table S3). The two-factor interaction model can be utilized to predict the DPPH radical scavenging capacity, and the equation is presented below (Equation (1)):

R1 (DPPH) = 305.62 − 23.20A − 128.95C + 78.43AC + 78.13C^2^ (1)

The ABTS radical scavenging capacity ranged from 531.94 to 2155.42 µmol Trolox/L. As in the DPPH analysis, the highest ABTS value was obtained at a pressure of 150 bar, a co-solvent concentration of 65%, and a flow rate of 0.5 mL/min (Table S1). ANOVA results revealed that pressure (A) had a significant effect on ABTS radical scavenging capacity (*p* = 0.0087), although its contribution was relatively weaker than that of other factors (Table S4). In contrast, flow rate (C) exhibited a highly significant and dominant effect on the response (*p* < 0.0001), indicating that it was the most influential single factor. In addition, the interaction between pressure and flow rate (AC) was highly significant (*p* = 0.0002), indicating that the effect of pressure on ABTS activity depends on the flow rate. Similarly, the quadratic term of flow rate (C^2^) was significant (*p* = 0.0003), indicating a nonlinear relationship between flow rate and antioxidant response. The reduced model exhibited a high coefficient of determination (R^2^ = 0.9037), explaining 90.37% of the variation in ABTS activity. The close agreement between the adjusted R^2^ (0.8716) and the R^2^ value confirms the adequacy of the model terms. The predicted R^2^ value (0.6682) indicates that the model has an acceptable predictive ability for new observations. The relatively low coefficient of variation (CV = 16.87%) indicates good precision and reliability in the experimental data. Furthermore, the adequate precision value of 18.9041 being well above the desirable threshold of 4, indicates an adequate signal-to-noise ratio and confirms that the model can reliably predict the response. The standard deviation and PRESS values were calculated to be 144.44 and 8.625 × 10^5^, respectively, further supporting the model’s overall predictive performance (Table S5). The regression equation for the model, which can be utilized for predicting the ABTS, is given below (Equation (2)):

R2 (ABTS) = 692.38 − 159.71A − 365.37C + 375.04AC + 348.47C^2^ (2)

The TPC of high-allicin garlic extract samples produced via the SC-CO_2_ extraction method was determined using the Folin–Ciocalteu method, and the results are presented in Table S1. The TPC ranged from 172.46 to 555.61 mg GAE/L. The highest TPC was determined in the extract obtained at a pressure of 150 bar, a co-solvent concentration of 65%, and a flow rate of 0.5 mL/min (Table S1). The overall model is statistically highly significant (*p* < 0.0001). When factors were evaluated individually, it was determined that the flow rate (C) had a highly potent and significant effect on the TPC response (*p* < 0.0001) (Table S6). The pressure × flow rate (AC) interaction was also found to be statistically significant (*p* = 0.0013). Conversely, pressure (A) alone did not provide a significant contribution to the model (*p* > 0.05). Upon examining the regression coefficients, it was determined that the linear term of flow rate (C), the pressure × flow rate interaction (AC), and the quadratic term of flow rate (C2) provided significant contributions to the TPC. These results demonstrate that the TPC response is particularly sensitive to flow rate and that the extraction yield of phenolic compounds is significantly influenced when evaluated in conjunction with pressure. The reduced model exhibited a high coefficient of determination (R^2^ = 0.9512), explaining 95.12% of the variation in total phenolic content. The close agreement between the adjusted R^2^ (0.9350) and the R^2^ value confirms the adequacy of the selected model terms. The predicted R^2^ value (0.8802) indicates a high predictive ability of the model for new observations. The relatively low coefficient of variation (CV = 9.12%) demonstrates a high level of precision and reliability in the experimental data. Furthermore, the adequate precision value of 26.0030, which is well above the desirable threshold of 4, indicates a strong signal-to-noise ratio and confirms that the response can be reliably predicted. The standard deviation and PRESS values were calculated as 28.85 and 24,524.34, respectively, further supporting the overall predictive performance of the model (Table S7). The regression equation for the model, which can be utilized for predicting the TPC, is given below (Equation (3)):

R3 (TPC) = +287.74 − 5.84A − 143.30C + 60.14AC + 60.99C^2^ (3)

It was determined that the allicin content of the extracts ranged from 1343.10 to 5745.55 mg/L. The highest allicin content was obtained under extraction conditions of 150 bar pressure, 65% (*v*/*v*) ethanol concentration, and a flow rate of 0.5 mL/min (Table S1). The overall reduced model was found to be statistically significant (*p* = 0.0006), indicating that the selected process variables significantly influenced the allicin content (Table S8). When the effects of the individual terms were examined, the flow rate (C) was identified as the most influential factor, showing a highly significant effect on allicin content (*p* = 0.0002). In addition, the interaction between pressure and flow rate (AC) significantly affected the response (*p* = 0.0386). In contrast, pressure (A) alone did not have a statistically significant effect on allicin content (*p* = 0.1839). Based on the regression coefficients and ANOVA results, the flow rate contributed most strongly to the model, while the pressure–flow rate interaction also played an important role in determining allicin extraction efficiency. Furthermore, the non-significant lack-of-fit value indicated that the model adequately described the experimental data. The model explained 72.59% of the variation in allicin content, with a coefficient of determination (R^2^ = 0.7259). The adjusted R^2^ value (0.6626) was reasonably close to the R^2^ value, indicating an acceptable model fit. However, the lower predicted R^2^ value (0.3527) suggests limited predictive capability for new observations. The relatively high coefficient of variation (CV = 30.69%) reflects substantial variability in the experimental data and lower experimental precision. Nevertheless, the adequate precision value of 12.3403, which is well above the desirable threshold of 4, indicates an adequate signal-to-noise ratio and demonstrates that the model can effectively distinguish between different response levels. The standard deviation and PRESS values were 697.71 and 1.495 × 10^7^, respectively (Table S9). Therefore, the developed model can be reliably used to predict allicin content, and the corresponding regression equation is presented below (Equation (4)).

R4 (Allicin content) = +2273.48 − 346.21A − 1285.71C + 802.51AC(4)

It was determined that the total sulfur compound (TSC) content ranged from 3485.88 to 16,395.20 mg/L. The highest amount of TSC was obtained under extraction conditions consisting of 150 bar pressure, 65% (*v*/*v*) ethanol concentration, and a flow rate of 0.5 mL/min (Table S1). The non-significance of the lack of fit term (*p* = 0.1361 > 0.05) indicates that the developed model is compatible with the experimental data and can be reliably used for prediction purposes. The overall model was statistically significant (*p* = 0.0002), indicating that the independent variables have a significant effect on the TSC (Table S10). Evaluation of the factors revealed that the flow rate (C) had a strong and statistically highly significant effect on the TSC content (*p* < 0.0001). The pressure × flow rate (AC) interaction was also found to be significant (*p* = 0.0226), indicating that jointly evaluating pressure and flow rate significantly influences the TSC response. Furthermore, the quadratic term of the flow rate (C^2^) was also identified as statistically significant (*p* = 0.0452), revealing that the TSC response exhibits nonlinear behavior with respect to the flow rate. Conversely, pressure (A) alone did not provide a significant contribution to the model (*p* = 0.2552). When the regression results are evaluated collectively, the total sulfur compound content is particularly sensitive to the flow rate, with both linear and quadratic effects playing decisive roles. The model exhibited a relatively high coefficient of determination (R^2^ = 0.8270), explaining 82.70% of the variation in the response variable. The close agreement between the adjusted R^2^ (0.7693) and the R^2^ value indicates that the model has a good fit and that the selected terms adequately describe the system. The predicted R^2^ value (0.4708), which is in reasonable agreement with the adjusted R^2^, suggests an acceptable, usable predictive capability for new observations. The coefficient of variation (CV = 23.48%) is at a reasonable level, indicating acceptable precision and reproducibility of the experimental data. Furthermore, the adequate precision value of 13.4774, well above the desirable threshold of 4, indicates a strong signal-to-noise ratio and confirms that the model can reliably discriminate among response variations (Table S11). The model can be utilized for the prediction of total sulfur compound content, and the corresponding regression equation is provided below (Equation (5)):

R5 (TSC) = +5918.55 − 667.34A − 3708.15C + 2064.28AC + 1715.27C^2^ (5)

Overall, the results indicate that a low flow rate is a decisive factor across all responses, while moderate pressure (150 bar) and 65% co-solvent concentration provide optimal extraction performance. Significant decreases in both antioxidant capacity and bioactive compound content were observed at high flow rates. These findings demonstrate that extraction yield can be optimized by balancing the interactions among pressure, co-solvent concentration, and, particularly, flow rate. The desirability function is a composite response index ranging from 0 to 1, which allows for the combination of multiple responses under a single optimization criterion; a value approaching 1 indicates that all specified target criteria are met simultaneously [35]. Within the scope of this study, as a result of the optimization based on the Box–Behnken experimental design developed using the RSM, the most suitable extraction conditions were determined via the desirability function approach as 150 bar pressure, 65% (*v*/*v*) co-solvent concentration, and a 0.5 mL/min flow rate. The determination of the optimum pressure as 150 bar can be explained by the fact that increasing pressure at specific temperatures enhances solvent density, thereby increasing the solubility of target compounds within the plant matrix. However, it has been reported that after solubility reaches a maximum at very high-pressure values, further pressure increases do not provide additional contributions to the extraction yield and may even lead to a decrease in some cases. The optimization of the co-solvent concentration at 65% can be attributed to the ethanol–water mixture, balancing the solubility of polar and semi-polar compounds. It is stated that in the SC-CO_2_ extraction of garlic, the ethanol–water ratio has a distinct but limited effect on extraction yield, and a slowdown or plateau in the extraction rate may occur as the solutes in the system reach equilibrium over time [36]. Furthermore, the optimization of the flow rate at 0.5 mL/min can be attributed to the increased contact time between the SC-CO_2_, co-solvent, and the plant matrix at lower flow rates. A longer contact time may have contributed to an increased extraction yield by facilitating the diffusion of target bioactive compounds from the matrix. These results demonstrate that optimum extraction conditions are determined by the balanced interaction between pressure, co-solvent concentration, and flow rate. To validate the developed optimization model, six independent extractions were performed under the optimum extraction conditions, and the obtained extracts were analyzed for all response parameters (R1–R5). The experimental values were compared with the corresponding predicted values generated by the model, and the percentage errors were calculated to assess predictive accuracy. The results demonstrated a strong agreement between predicted and experimental values, with percentage errors ranging from 1.05% to 6.67%. Furthermore, all experimental values were found to lie within the 95% prediction intervals of the model, confirming its adequacy and robustness. The high correlation between predicted and experimental responses (R^2^ = 0.9964) further supports the reproducibility and predictive capability of the developed optimization model. Within the scope of our study, the purity of allicin in the supercritical extraction process was determined to be 45.77%.

#### 3.1.2. Optimization of MD Conditions and Purification of Allicin Using High-Allicin Garlic Extract Obtained by SC-CO_2_ Extraction

**Figure 2:**
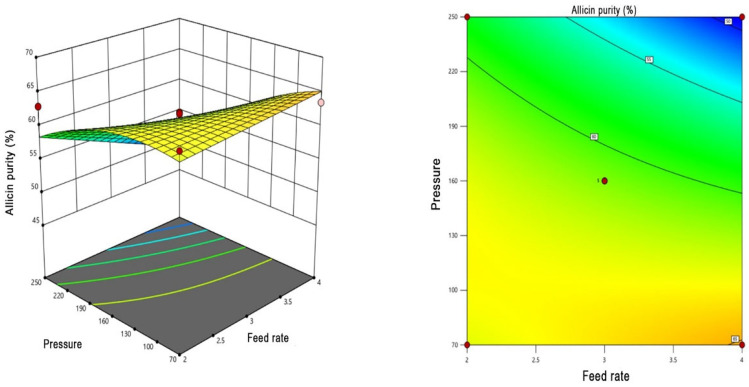
Contour and response surface plots obtained during the molecular distillation process.

Molecular distillation, or more specifically short-path vacuum evaporation, leverages reduced pressure conditions to decrease the boiling points of thermolabile compounds, thereby minimizing heat exposure time and preventing thermal degradation. In a short-path distillation process, the pressure inside the evaporation unit can be reduced up to 10^−3^ mbar, and the practical vacuum level depends on the volatile nature of the target matrix and the specific limits of the processing equipment. Therefore, operating at the selected vacuum levels in this study can be considered sufficient to effectively separate and purify sensitive thiosulfinates like allicin without triggering structural transformation into secondary sulfides [37,38]. This technique is widely utilized for the purification of thermally unstable compounds characterized by low volatility and high viscosity. In this context, molecular distillation offers a suitable and effective approach for the high-purity recovery of allicin obtained via SC-CO_2_ extraction. Following the initial optimization, the extract obtained under optimal SC-CO_2_ conditions (150 bar pressure, 65% (*v*/*v*) co-solvent concentration, and a 0.5 mL/min flow rate) was further processed and optimized in the MD unit using a Box–Behnken design within RSM. The ANOVA results for the reduced model developed according to the Box–Behnken experimental design are presented in Table S13. The contour and response surface plots obtained during the molecular distillation process are shown in Figure 2. The overall model was determined to be statistically highly significant (*p* < 0.0001), indicating that the selected process parameters (wiper speed, feed rate, pressure, evaporator temperature, and condenser temperature) have substantial effects on allicin purity (Table S12). When the factors were evaluated individually, it was determined that pressure (C) and evaporator unit temperature (D) exhibited highly significant effects on allicin purity (*p* < 0.0001). The feed rate (B) was also found to be statistically significant (*p* = 0.0025) (Table S13). Conversely, it was determined that the wiper speed (A) and condenser temperature (E) did not exhibit significant effects individually (*p* > 0.05). Upon examining the interaction terms, the interactions between wiper speed and evaporator temperature (AD), and pressure and evaporator temperature (CD) were found to be statistically significant (*p* < 0.05). These findings reveal that allicin purity in the MD process is primarily determined by the evaporator temperature and pressure, while the feed rate has a secondary influence. Consequently, the co-optimization of evaporator temperature and pressure is of critical importance for achieving high allicin purity. The model has a high coefficient of determination (R^2^ = 0.8724), explaining 87.24% of the variation in allicin purity. The adjusted R^2^ value (0.8257) is close to the R^2^ value, indicating that the model fits the data well and that the selected terms adequately explain the variation in the data. The fact that the predicted R^2^ value (0.6566) is consistent with the adjusted R^2^ demonstrates that the model possesses an acceptable and reliable predictive power for new observations. The very low coefficient of variation (3.51%) indicates that the experimental data exhibit high precision and reproducibility. Furthermore, the Adeq Precision value of 16.5620, which is well above 4, indicates that the model possesses a strong signal-to-noise ratio and can reliably distinguish changes in the response (Table S14). The developed model can be utilized to predict allicin purity, and the regression equation is provided below (Equation (6)):

R6 (Allicin purity) = +61.26 − 0.7774A − 1.85B − 5.21C + 6.11D − 0.4465E − 2.87BC + 2.81BD + 7.32CD − 2.38C^2^ − 2.42D^2^ + 1.54E^2^ (6)

The MD conditions optimized using the desirability function approach are as follows: a wiper speed of 167.129 rpm, a feed rate of 2.025 mL/min, a pressure of 243.567 mbar, an evaporator unit temperature of 79 °C, and a condenser temperature of 10 °C (Table S7). The desirability value obtained as a result of this optimization study indicates that the specified criteria were largely satisfied. This approach aimed to both maximize allicin purity and enhance process efficiency by determining the most suitable combination of process parameters. Allicin purity (45.59–69.19%) was significantly influenced by the process parameters. The results demonstrate that the feed rate, pressure, and evaporator temperature are particularly critical factors. A low feed rate (2 mL/min) was generally associated with higher purity values, which can be attributed to the increased residence time in the system and more effective fractionation. Similarly, low-pressure conditions (especially 70 mbar) tended to increase allicin purity. Increasing the evaporator temperature to 80 °C also positively influenced purity, whereas lower purity values were obtained at 30 °C. Regarding the wiper speed, 160 rpm was observed in most high-purity instances, suggesting that a moderate speed provides optimum conditions. Furthermore, maintaining the condenser temperature within the range of 10–15 °C yielded more advantageous results. The highest allicin purity was achieved with a combination of 160 rpm wiper speed, 2 mL/min feed rate, 160 mbar pressure, 80 °C evaporator temperature, and 15 °C condenser temperature. These findings indicate that the concurrent application of low pressure, low feed rate, and high evaporator temperature is more effective for allicin purification. Within the scope of this study, the optimum extraction conditions were determined using a desirability function approach based on a Box–Behnken experimental design developed via RSM. The MD conditions optimized using the desirability function approach were identified as follows: a wiper (scraper) speed of 167.129 rpm, a feed rate of 2.025 mL/min, a pressure of 243.567 mbar, an evaporator temperature of 79 °C, and a condenser temperature of 10 °C. The desirability value obtained from this optimization indicates that the specified criteria were largely satisfied. This approach aimed to both maximize allicin purity and improve process efficiency by determining the optimal combination of process parameters. Validation of the optimized conditions was performed to evaluate the reproducibility and reliability of the obtained results. Under optimal conditions, six independent MD runs were conducted, and the analysis showed that the actual allicin purity was 67.10, while the predicted value was 68.32. In the supercritical extraction process, the purity of allicin was 45.77%, which increased to 69.19% after molecular distillation. A study exists in the literature regarding allicin purification via MD, in which the purification of allicin-rich garlic extract obtained through SC-CO_2_ extraction was investigated in detail. In the study, the extract obtained via SC-CO_2_ was subjected to a short-path molecular distillation system, and the effects of pressure, evaporator temperature, and feed rate on allicin purity and recovery were specifically evaluated. It was reported that low-pressure conditions enabled separation at lower temperatures by reducing the boiling point of allicin, thereby minimizing thermal degradation. A controlled increase in evaporator temperature facilitated the selective evaporation of allicin while retaining lower-volatility impurities in the system. As a result of multi-stage molecular distillation, it was reported that the allicin concentration increased gradually, reaching a purity level of 68% (*w*/*w*) in the final product [16]. Unlike previous studies, this work involves the use of Design-Expert software to carry out comprehensive optimizations in both supercritical fluid extraction and molecular distillation processes. Furthermore, by optimizing the purity and yield of the product, a more efficient and controlled purification process has been achieved. For the first time in literature, the bioactive properties of the obtained products have been examined in detail, with the bioactive potential of allicin in particular being comprehensively evaluated. These findings demonstrate that the combination of SC-CO_2_ extraction and molecular distillation enables high-purity production while preserving the stability of allicin, a heat-sensitive and reactive compound. Furthermore, this approach offers an industrially applicable process strategy for food, nutraceutical, and pharmaceutical applications.

### 3.2. Characterization and Bioactivity Analyses of Allicin-Rich Garlic Extract Obtained from SC-CO_2_ and SC-CO_2_-MD

#### 3.2.1. Physicochemical Properties of Garlic Extracts

The physicochemical analysis results for the garlic extract, produced under optimum conditions via SC-CO_2_ extraction and characterized by high allicin content and bioactive properties, are presented in Table 2. The L* value of the extract obtained under optimum conditions was determined to be 94.60, indicating a very high degree of lightness. An a* value of −3.65 indicates a shift toward green tones, while a b* value of 15.43 reveals the dominance of yellowness. Based on these results, the garlic extract obtained via SC-CO_2_ extraction was characterized by a light appearance with yellow-green undertones.

The formation mechanism of the yellow color in garlic is attributed to its sulfur compounds and their respective reaction pathways. Upon crushing or chopping garlic, cellular integrity is disrupted, allowing alliin to react with the alliinase enzyme to form various organosulfur compounds. These compounds interact with pigment precursors, leading to the manifestation of yellow hues in garlic [39]. In garlic extracts with high allicin content, the pH value is another critical parameter. The pH of the extract obtained via SC-CO_2_ extraction was determined to be 6.53. Following its formation, allicin can transform into various secondary compounds depending on the ambient temperature and pH [40]. Furthermore, it has been reported that pH exerts a significant effect on allicin stability, with lower pH values favoring an extended shelf life [41]. The physicochemical properties of the SC-CO_2_-MD sample, produced by the purification of the extract obtained via the SC-CO_2_ method using MD, are presented in Table 2. The pH value of the SC-CO_2_-MD sample was determined to be 7.67, indicating that the extract possesses slightly basic (alkaline) properties. A light-colored extract was obtained with an L* value of 95.50, while the negative a* value (−2.57) indicates the dominance of greenish tones. These color variations are associated with the sulfur and phenolic content of the extract; the high L* value suggests reduced polyphenol oxidation and minimal melanoidin formation [42,43]. The SC-CO_2_ method facilitates the more intensive extraction of fat-soluble bioactive components due to its capacity to dissolve non-polar compounds [44].

#### 3.2.2. Antioxidant Potential

Allicin is known to possess a more potent antioxidant activity compared to many other chemical components found in garlic [45,46,47]. Allicin protects cells against oxidative stress by stimulating the production of antioxidant compounds, reducing the formation of cytotoxic substances, and contributing to the scavenging of free radicals. Additionally, it has been reported that this antioxidant potential may be beneficial in the prevention of various cardiovascular diseases [48]. Within the scope of this study, the antioxidant capacity of garlic extracts with high allicin content, obtained via SC-CO_2_ and SC-CO_2_-MD, was determined using DPPH, ABTS, CUPRAC, and FRAP methods.

The DPPH, ABTS, CUPRAC, and FRAP values of garlic extracts obtained via SC-CO_2_ extraction were determined as 621.85 µmol Trolox/L, 2008.50 µmol Trolox/L, 5.27 mM Trolox/L, and 181.81 mg Trolox/L, respectively. Dinu et al. [49] examined various garlic varieties and reported the highest antioxidant capacities determined by the DPPH and ABTS methods as 1.12 and 1.41 µmol Trolox/g, respectively. Similarly, Ciric et al. [50] identified the highest antioxidant capacity measured via the CUPRAC method as 3.75 mmol Trolox/g in a garlic sample cultivated in Spain. The TPC of the garlic extracts obtained through SC-CO_2_ extraction was determined to be 595.99 mg GAE/L. SC-CO_2_-MD increased allicin purity; however, antioxidant assays and total phenolic content decreased significantly compared to the SC-CO_2_ extract. This suggests that some bioactive compounds may have been removed during the purification process in the MD operation. In a study conducted by Chhouk et al. [51], to obtain phenolic compounds from garlic husks, extraction was performed using CO_2_-expanded ethanol at a pressure of 10 MPa, a temperature range of 50–200 °C, and a CO_2_ flow rate of 0.5–2 mL/min; the TPC of dried garlic husks was reported as 56.26 mg GAE/g. Cavalcanti et al. [52] compared various solvents and extraction techniques to determine bioactive compounds and antioxidant activity in garlic. They determined the TPC of the extract obtained using ultrasound-assisted extraction (UAE) and ethanol as 0.84 mg GAE/g. Furthermore, Ciric et al. [50], detected the highest TPC value in Chinese garlic at 25.57 mg GAE/g among different garlic samples. The antioxidant capacity of the SC-CO_2_-MD sample was measured via DPPH, ABTS, CUPRAC, and FRAP methods as 32.15 µmol Trolox/L, 270.01 µmol Trolox/L, 0.12 mM Trolox/L, and 102.36 mg Trolox/L, respectively (Table 2). The total phenolic content (TPC) of the SC-CO_2_-MD sample was determined to be 117.90 mg GAE/L. During MD, allicin can transform into more stable sulfur compounds (diallyl disulfide, diallyl trisulfide, ajoene, etc.) depending on variations in oxygen, heat, and pH, thereby contributing to the total antioxidant capacity. Additionally, phenolic and organosulfur compounds enhance the extract’s antioxidant potential by neutralizing reactive oxygen species (ROS) [53,54].

#### 3.2.3. Antimicrobial Activity

The antimicrobial activity of the garlic extract obtained via the SC-CO_2_ method was determined using the agar well diffusion method, and the results are presented in Table 3. The inhibition zone diameters of the SC-CO_2_ extract for *E. coli*, *S. aureus*, *B. subtilis*, *P. aeruginosa*, and *K. pneumoniae* were identified as 35.67, 44.17, 32.33, 28.33, and 28.83 mm, respectively (Table 3). In the literature, allicin has been reported to be effective against both Gram-positive and Gram-negative bacteria, inhibiting the growth of pathogens such as *Bacillus* spp., *Streptococcus* spp., *Salmonella typhimurium*, *Agrobacterium tumefaciens*, *Escherichia coli*, *Candida albicans*, *Vibrio cholerae*, and *Pseudomonas syringae* [55,56,57]. Within the scope of this study, the minimum inhibitory concentration (MIC) of the garlic extract obtained via the SC-CO_2_ method was determined. In the analyses, the effects of eight different concentrations of the garlic extract obtained via the SC-CO_2_ method, namely 1.45, 0.72, 0.36, 0.18, 0.09, 0.04, 0.02, and 0.01 mg/mL, on the growth of *E. coli*, *S. aureus*, *B. subtilis*, *K. pneumoniae*, and *P. aeruginosa* were investigated. It was determined that the extract inhibited the growth of *E. coli* during the incubation period within the concentration range of 0.01–1.45 mg/mL, and the MIC was identified as 0.04 mg/mL. In the literature, allicin has been reported to exhibit MIC values in the range of 32–64 µg/mL against microorganisms such as *S. aureus*, *E. coli*, *Acinetobacter baumannii*, and *Candida albicans* [58]. In the same study, the MIC value for the *E. coli* MG1655 strain was determined to be 23 µg/mL (0.023 mg/mL). However, in another study, the MIC values of garlic extract were reported as 16 mg/mL for *E. coli* and *P. aeruginosa*, 32 mg/mL for *S. marcescens* and MRSA, and 64 mg/mL for *K. pneumoniae* [59]. It was determined that the extract inhibited bacterial growth in the concentration range of 0.01–1.45 mg/mL, and the MIC value for *S. aureus* was found to be 0.04 mg/mL. In the literature, it has been reported that allicin can exhibit potent antimicrobial activity against *S. aureus* at a level of 8 µg/mL [60]. Regarding its effect on *K. pneumoniae*, it was determined that the extract suppressed bacterial growth within the concentration range of 0.01–1.45 mg/mL, with an MIC value of 0.04 mg/mL. *K. pneumoniae* is considered a significant pathogen in hospital-acquired infections, particularly due to increasing antibiotic resistance [61]. When its effect on *B. subtilis* was evaluated, it was identified that the garlic extract obtained via the SC-CO_2_ method inhibited bacterial growth within the concentration range of 0.01–1.45 mg/mL, with an MIC value of 0.04 mg/mL. *B. subtilis* is a spore-forming, Gram-positive bacterium frequently isolated as a spoilage agent, particularly in food products [62]. The effect on *P. aeruginosa* was evaluated, and it was determined that the extract inhibited bacterial growth in the concentration range of 0.01–1.45 mg/mL, with an MIC value of 0.09 mg/mL. *P. aeruginosa* is an opportunistic pathogen capable of exhibiting resistance to multiple antibiotics and plays a crucial role, especially in hospital-acquired infections [63]. The results demonstrate that the garlic extract with high allicin content, produced via the SC-CO_2_ method, possesses strong antimicrobial activity against both Gram-positive and Gram-negative bacteria.

The SC-CO_2_-MD sample exhibited significant inhibition against bacterial strains, including *E. coli* (27.51 mm), *S. aureus* (37.53 mm), and *B. subtilis* (31.56 mm). Among the tested bacteria, the strongest inhibitory activity was observed against *S. aureus* and *B. subtilis*, whereas relatively lower inhibition zones were recorded for *P. aeruginosa* (12.55 mm) and *K. pneumoniae* (20.73 mm). This situation is attributed to the resistance of Gram-negative cells to the entry of compounds such as allicin, due to the lipopolysaccharide (LPS) layer in their outer membrane and the presence of efflux pumps [64]. The MIC of the SC-CO_2_-MD sample was determined against *E. coli*, *S. aureus*, *B. subtilis*, *K. pneumoniae*, and *P. aeruginosa* using allicin concentrations ranging from 1.11 to 0.01 mg/mL. The MIC values revealed differences in bacterial susceptibility. The MIC value for *E. coli* was determined to be 0.07 mg/mL, indicating that the sample exhibits significant inhibitory activity even against Gram-negative bacteria. For *S. aureus* and *K. pneumoniae*, the MIC values were identified as 0.07 mg/mL, demonstrating that SC-CO_2_-MD is effective against these Gram-positive and Gram-negative bacteria. The MIC value for *B. subtilis* was determined as 0.06 mg/mL, reflecting high susceptibility among Gram-positive bacteria. Conversely, *P. aeruginosa* exhibited a higher MIC value (0.14 mg/mL) compared to the other bacteria, suggesting a relatively lower inhibitory potential of the sample against this specific pathogen. Overall, the SC-CO_2_-MD sample exhibited a concentration-dependent inhibitory effect on all tested bacteria. While it was effective at lower concentrations against Gram-positive bacteria (*S. aureus* and *B. subtilis*), varying levels of susceptibility were observed among Gram-negative bacteria (*E. coli*, *K. pneumoniae*, and *P. aeruginosa*). These findings demonstrate that the SC-CO_2_-MD sample possesses broad-spectrum antimicrobial properties and exhibits high inhibitory potential, particularly against Gram-positive bacteria. The antimicrobial effect of allicin is associated with its ability to readily penetrate microbial cells and rapidly diffuse through phospholipid membranes due to its lipophilic nature [65]. Furthermore, allicin reacts with free thiol groups in proteins, specifically interacting with cysteine and glutathione (GSH); it reduces cellular GSH levels by converting GSH into S-allylmercaptoglutathione (GSSA), thereby inducing oxidative stress [66]. Additionally, it inhibits essential enzymes by inducing S-thioallylation in proteins and can disrupt cellular signaling pathways [58]. Allicin has also been reported to inhibit DNA, RNA, and protein synthesis; notably, its impact on RNA synthesis plays a critical role in arresting bacterial growth. Due to its volatility, allicin can exhibit antibacterial and antifungal activity in the gas phase and was historically used in the treatment of tuberculosis infections caused by *Mycobacterium tuberculosis* [67,68]. In the SC-CO_2_-MD sample purified via MD, the stability of allicin and other heat-sensitive bioactive compounds was maintained, providing effective inhibition against Gram-positive and specific Gram-negative bacteria.

#### 3.2.4. Antifungal Activity

The antifungal activity of garlic has been compared to that of the reference antimycotic drug, fluconazole [69]. Similarly, garlic extracts have been reported to inhibit the growth of pathogenic fungi such as *Botrytis cinerea*, *Penicillium expansum*, *Neofabraea alba*, *Fusarium*, and *Rhizopus species* [70]. The broad-spectrum antifungal activity of allicin has also been validated against various phytopathogenic fungi. Antifungal activity tests were conducted against mold species, including *A. fumigatus* (ATCC^®^ 204305™), *P. chrysogenum* (ATCC^®^ 10106™), *C. cladosporioides* (ATCC^®^ 16022™), and *Alternaria* (ATCC^®^ 20084™), with nystatin utilized as a positive control.

The inhibition zone diameters generated by nystatin ranged from 20.07 mm to 22.39 mm. These results indicate that the tested mold species are susceptible to nystatin and demonstrate that this drug was appropriately utilized as a standard antifungal agent in the study. Extracts obtained via the SC-CO_2_ method exhibited larger inhibition zone diameters (29.12–29.38 mm) than nystatin for most mold species. This indicates that the antifungal activity of the extracts obtained with SC-CO_2_ is remarkably potent. Notably, the highest inhibition zone of 29.38 mm was observed for *A. fumigatus* and *Alternaria species*. Conversely, the inhibition zone diameter for *C. cladosporioides* was determined to be 18.74 mm, which was found to be lower than that of nystatin. The high efficacy of the SC-CO_2_ extraction method in terms of antifungal activity can be attributed to its capacity to extract lipophilic compounds with high purity and efficiency. It is well-established that organosulfur compounds in garlic, particularly allicin, possess strong antifungal activity [69]. The findings of this study reveal that SC-CO_2_ extraction is an effective method for obtaining natural antifungal agents from garlic and that the resulting extracts can exhibit significant antifungal activity against various mold species. In the study, it was determined that the allicin-containing SC-CO_2_-MD sample exhibited antifungal activity against selected mold species. The highest inhibition zone was recorded for *A. fumigatus* (27.51 mm), and the lowest inhibition zone of 11.56 mm was determined for *C. cladosporioides*. The primary antifungal mechanism of allicin involves disrupting membrane integrity by inducing structural changes in ergosterol within the fungal cell membrane, which leads to the leakage of cellular contents [71]. This effect is particularly pronounced in *Penicillium* and *Aspergillus* species. The extensive inhibition zone of 27.51 mm observed for *A. fumigatus* in this study can be attributed to the disruption of cell membrane integrity by allicin. Furthermore, allicin can cause mitochondrial dysfunction and trigger apoptosis (programmed cell death) mechanisms by increasing the generation of reactive oxygen species (ROS) [72]. Conversely, the 11.56 mm inhibition zone observed for *C. cladosporioides* suggests that this species may be relatively more resistant to antifungal compounds. The presence of complex polyphenolic compounds within the cell wall structure of *C. cladosporioides* may contribute to the development of higher resistance against antifungal agents [73].

#### 3.2.5. Determination of Bioavailability Using an In Vitro Digestive System

Digestion models are essential tools for evaluating the post-digestive behavior and bioaccessibility potential of foods by mimicking human digestive processes. Specifically, in vitro digestion models provide an opportunity to examine the changes that food components undergo during digestion, as well as their bioaccessibility and potential metabolic effects under laboratory conditions [74]. In this study, the INFOGEST static in vitro digestion model, adapted for older adults, was utilized to determine the bioaccessibility of high-allicin garlic extracts. Consequently, variations in antioxidant capacity, TPC, allicin, and TSC were evaluated under digestive conditions representing younger adult (<65 years) and older adult (>65 years) individuals [33]. The widely used DPPH, ABTS, CUPRAC, and FRAP methods reveal different mechanisms of antioxidant capacity. Evaluation of the antioxidant capacity results showed that for the younger adult digestion model, the DPPH values for the oral, gastric, and intestinal phases were 614.10, 902.00, and 85.80 μmol Trolox/L, respectively; ABTS values were 1964.12, 2366.45, and 174.72 μmol Trolox/L; CUPRAC values were 1.83, 2.62, and 0.27 mM Trolox/L; and FRAP values were 171.50, 188.73, and 80.59 mg Trolox/L. In the older adult digestion model, the corresponding values were 588.38, 869.98, and 95.83 μmol Trolox/L (DPPH); 1978.70, 2583.60, and 161.46 μmol Trolox/L (ABTS); 1.84, 2.28, and 0.15 mM Trolox/L (CUPRAC); and 169.47, 186.67, and 65.90 mg Trolox/L (FRAP). Upon examining the TPC of the extracts obtained via the SC-CO_2_ method, the values for the oral, gastric, and intestinal phases in the younger adult model were 516.79, 587.83, and 183.55 mg GAE/L, respectively, while in the older adult model, they were 515.62, 582.08, and 179.49 mg GAE/L. The results indicated that the highest TPC was observed in the gastric phase in both models. Overall, it was determined that both antioxidant capacity and total phenolic content peaked in the gastric phase, followed by the oral and intestinal phases. This trend is thought to be associated with the release of phenolic compounds during digestion and the subsequent increase in their bioaccessibility. Upon examining the allicin content, it was determined as 5008.31 mg/L, 3593.29 mg/L, and 172.71 mg/L in the oral, gastric, and intestinal phases of the young adult model, respectively, whereas in the older adult model, these values were 4917.48 mg/L, 3749.76 mg/L, and 180.00 mg/L. It is well-established that the stability of allicin is highly sensitive to pH. Specifically, under the acidic conditions of the gastric environment, allicin becomes unstable and may undergo degradation [74]. Indeed, it has been reported that allicin is more stable within the pH range of 5–6, but remains highly unstable at conditions below pH 1.5 and above pH 11 [41]. This indicates that the bioaccessibility of allicin can be significantly influenced by pH fluctuations within the digestive environment.

Allicin, a prominent organosulfur compound in garlic, is known to play a vital role in the antioxidant capacity of garlic extracts. It is reported that allicin can effectively scavenge free radicals, thereby contributing to the total antioxidant capacity of garlic extracts [75]. Furthermore, a positive correlation exists between total phenolic content and antioxidant capacity, and high TPC values are associated with increased antioxidant indices [76].

The study results demonstrated that allicin levels decreased significantly throughout the digestion process due to the influence of pH and enzymatic activities. While allicin remained largely stable in the oral phase, characterized by a neutral pH, substantial degradation occurred in the gastric phase due to the low pH. In the intestinal phase, allicin levels were found to have dropped to very low concentrations. While the sequential SC-CO_2_ extraction and short-path vacuum distillation matrix successfully accomplished a massive upgrade in initial chemical purity and guaranteed stable, elevated bioaccessibility specifically within the acidic gastric window, the subsequent intestinal annihilation confirms that high structural processing purity does not automatically translate into sustained systemic bioaccessibility of the intact molecule. This physiological termination underscores the acute necessity to declare the fundamental biological limitations of free allicin delivery. The rapid nucleophilic breakdown driven by the alkaline shift (pH 7.0–7.5) and the complex pancreatin enzymatic environment inside the duodenum accelerates the self-condensation of the diallyl thiosulfinate core into lipophilic volatile sulfides. Consequently, there is an imperative engineering need to implement targeted encapsulation or matrix stabilization strategies. Enveloping this purified organosulfur fraction within pH-responsive biopolymer architectures, such as alginate-pectin hydrogel networks, liposomal entrapment vectors, or core–shell microencapsulation matrices utilizing spray-chilling methodologies, represents the critical missing link. These advanced delivery systems are mandatory to physically shelter the volatile thiosulfinate linkage against premature duodenal ionization, effectively delaying the release kinetics until the bioactive core reaches distal epithelial absorption zones. In conclusion, it was determined that garlic extracts obtained via the SC-CO_2_ method exhibit high TPC and antioxidant activity during the in vitro gastrointestinal digestion process, particularly in the gastric phase. This is attributed to the extract’s high allicin content, the bioaccessibility of phenolic compounds, and the overall impact of digestive conditions. Studies in the literature also indicate that in vitro gastrointestinal digestion can significantly influence the phenolic composition and antioxidant capacities of plant extracts [77,78]. Similarly, several researchers have reported that the digestion process can lead to changes or increases in antioxidant activity by enhancing the bioaccessibility of phenolic compounds [79,80]. The bioaccessibility of the SC-CO_2_-MD sample was measured across the oral, gastric, and intestinal phases, and the variations between young and older adults were compared. The DPPH and ABTS values measured in the gastric phase post-in vitro digestion indicate that the free radical scavenging potential of allicin and other phenolic compounds reaches its peak in the gastric environment. Similarly, CUPRAC and FRAP values were also at their maximum in the gastric phase, whereas the values in the oral and intestinal phases were found to be significantly lower (Table 4). These findings demonstrate that allicin and other bioactive compounds become more bioaccessible within the gastric environment and that the SC-CO_2_-MD extract can exhibit potent antioxidant efficacy, even specifically in older adults. As shown in Table 4, the highest allicin content in the SC-CO_2_-MD sample was detected during the oral phase, determined as 927.71 mg/L for young adult and 923.80 mg/L for the older adults. As the digestion process progressed, a significant decrease in allicin content was observed; values of 762.56 mg/L in young adults and 719.11 mg/L in the older adults were measured during the gastric phase. Allicin was undetectable in the intestinal phase, which was evaluated as an indication that allicin was significantly decomposed in the gastric environment and underwent complete degradation in the intestine. Differences observed between young and older adults are particularly prominent in the gastric phase, arising from changes in gastric pH and enzyme activity in older adults. These findings reveal that allicin is an unstable compound within the gastrointestinal environment and undergoes rapid degradation, especially under acidic gastric conditions. In alignment with the literature, the pH sensitivity of allicin directly affects its bioaccessibility, and its degradation in the gastric environment limits its reaching the bloodstream and other tissues [41,74]. Overall, in vitro digestion studies on the SC-CO_2_-MD sample demonstrate that although allicin is present in high amounts during the oral phase, it significantly decreases during the gastric phase and remains undetectable in the intestine; this reveals that allicin stability is limited by pH and enzymatic effects, leading to low bioaccessibility.

Overall, when evaluated using the in vitro gastrointestinal digestion model, the SC-CO_2_-MD sample is characterized by high initial levels of allicin and phenolic compounds and exhibits maximum antioxidant capacity in the gastric phase. However, the decomposition of allicin in the gastric environment, leading to its being undetectable in the intestine and the loss of antimicrobial efficacy, demonstrates that this compound is unstable within the gastrointestinal tract and that its bioaccessibility may be limited.

#### 3.2.6. The Effect of In Vitro Gastrointestinal Digestion on Antimicrobial Activity

Antimicrobial activity analyses of samples taken from the oral, gastric, and intestinal phases of garlic extracts obtained via the SC-CO_2_ method were determined using the agar well diffusion method, and the resulting inhibition zone diameters are provided in Table 4. According to the results, for *E. coli*, inhibition zones of 26.50 mm and 26.00 mm were observed in the oral and gastric phases of the young adult digestion model, respectively, while these values were determined as 25.83 mm and 21.50 mm in the older adult model. For the *S. aureus* strain, inhibition zones of 36.33 mm and 31.33 mm were measured in the oral and gastric phases of the young adult model, while 34.67 mm and 31.33 mm were recorded in the older adult model, respectively. For *B. subtilis*, inhibition zones of 17.50 mm and 14.50 mm were identified in the oral and gastric phases of the young adult model, and 18.50 mm and 11.50 mm in the older adult model. In the *P. aeruginosa* strain, inhibition zones of 23.00 mm and 24.17 mm were detected in the oral and gastric phases of the young adult model, while 23.50 mm and 26.00 mm were found in the older adult model, respectively. For the *K. pneumoniae* strain, inhibition zones of 13.67 mm and 13.83 mm were measured in the oral and gastric phases of the young adult model, whereas 14.17 mm and 11.67 mm were recorded in the older adult model. No inhibition was observed in the intestinal phase in either digestion model. These findings indicate that the antimicrobial activity of garlic extracts can vary throughout the digestion process. While significant antimicrobial activity was observed particularly in the oral and gastric phases, it was determined that the activity disappeared in the intestinal phase. This situation is thought to be associated with the degradation of allicin, the primary bioactive compound of garlic, due to pH variations and enzymatic activities during digestion. Specifically, it is evaluated that the low pH conditions in the gastric environment may reduce allicin stability, leading to a significant decrease in antimicrobial efficacy upon reaching the intestinal phase. The minimum inhibitory concentration (MIC) was determined for the SC-CO_2_ samples exhibiting antimicrobial activity. For *E. coli*, the young gastric phase of SC-CO_2_ inhibited bacterial growth within an allicin concentration range of 0.005–0.72 mg/mL, with the lowest MIC value determined as 0.04 mg/mL. The MIC was identified as 0.04 mg/mL in the older adult oral phase and 0.09 mg/mL in the older adult gastric phase. For *S. aureus*, the MIC values were determined as 0.04 mg/mL for the young oral phase, 0.09 mg/mL for the young gastric phase, 0.04 mg/mL for the older adult oral phase, and 0.09 mg/mL for the older adult gastric phase. For *K. pneumoniae*, the MIC was found to be 0.04 mg/mL in the oral phases and 0.09 mg/mL in the gastric phases. Similarly, in *B. subtilis*, the MIC was identified as 0.04 mg/mL for the oral phases and 0.09 mg/mL for the gastric phases. For *P. aeruginosa*, the MIC value was determined as 0.18 mg/mL for all active oral and gastric phases. In conclusion, garlic extracts obtained via SC-CO_2_ exhibited potent antimicrobial activity in the oral and gastric phases of both young and older adult digestion models. The determined MIC values ranged from 0.04 to 0.18 mg/mL, depending on the bacterial species. No significant difference was observed in terms of MIC between the young and older adult models. These results indicate that SC-CO_2_ garlic extracts retain antimicrobial activity mainly during the oral and gastric phases; however, this activity was not maintained in the intestinal phase. Furthermore, SC-CO_2_-MD samples showed no detectable antimicrobial activity following digestion. Regarding antimicrobial activity, it was determined that the SC-CO_2_-MD sample exhibited no inhibitory effect against *E. coli*, *S. aureus*, *B. subtilis*, *P. aeruginosa*, and *K. pneumoniae* strains in the oral, gastric, and intestinal phases following in vitro digestion. This indicates that the digestion process reduces the microbial activity of allicin, resulting in the loss of the extract’s antimicrobial effect under gastric and intestinal conditions.

Overall, when evaluated using the in vitro gastrointestinal digestion model, the SC-CO_2_-MD sample is characterized by high initial levels of allicin and phenolic compounds and exhibits maximum antioxidant capacity in the gastric phase. However, the decomposition of allicin in the gastric environment, resulting in it being undetectable in the intestine and the subsequent loss of antimicrobial efficacy, demonstrates that this compound is unstable within the gastrointestinal tract and that its bioaccessibility may be limited. For the SC-CO_2_-MD sample, digestion resulted in complete loss of antimicrobial activity, whereas SC-CO_2_ extracts retained activity during the oral and gastric phases but not in the intestinal phase.

#### 3.2.7. DNA-Protective Effect

**Figure 3:**
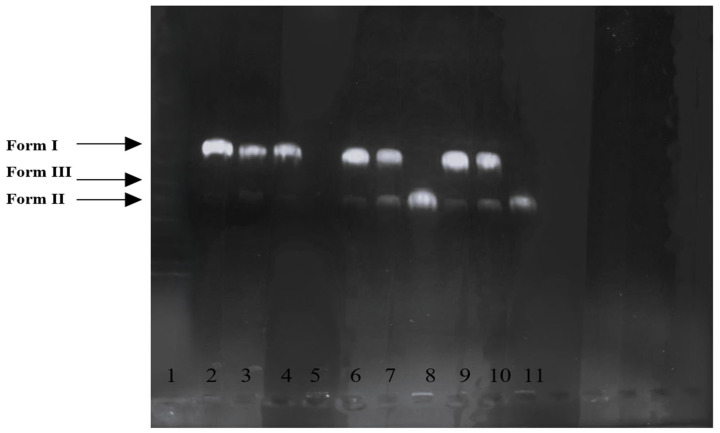
Gel images illustrating the protective effects of the samples on plasmid DNA against OH radicals generated by the photolysis of H_2_O_2_. Lanes: 1: Marker, 2: Plasmid DNA, 3: Plasmid DNA + UV, 4: Plasmid DNA + H_2_O_2_, 5: Plasmid DNA + UV + H_2_O_2_, 6: SC-CO_2_ + Plasmid DNA + H_2_O_2_, 7: SC-CO_2_ + Plasmid DNA + UV, 8: SC-CO_2_ + Plasmid DNA + H_2_O_2_ + UV, 9: SC-MD + Plasmid DNA + H_2_O_2_, 10: SC-MD + Plasmid DNA + UV, 11: SC-MD + P Plasmid DNA + H_2_O_2_ + UV.

Allicin possesses a protective role in the body due to its antioxidant, anti-inflammatory, antihypertensive, and cardiovascular effects [81]. As an antioxidant phytochemical, it scavenges reactive oxygen species and protects cells against oxidative DNA damage [82]. In this study, the protective effect of garlic extracts produced via SC-CO_2_ extraction under optimum conditions against oxidative damage to pBR322 plasmid DNA, induced by the UV photolysis of H_2_O_2_, was evaluated using agarose gel electrophoresis. The natural supercoiled form (Form I), the open circular form (Form II) containing single-strand breaks, and the linear form (Form III) containing more extensive breaks were observed. In control experiments (lane 5), the DNA exposed to UV in the presence of H_2_O_2_ was completely degraded, and it was determined that hydroxyl (OH∙) radicals caused strand breaks in the DNA structure. In samples where the SC-CO_2_ extract was added, while the supercoiled form (Form I) of the DNA could not be preserved, a distinct effect was observed in the preservation of the open circular (Form II) and linear (Form III) forms. This result indicates that the SC-CO_2_ extract protects specific structural forms of DNA against OH∙ radicals originating from the UV photolysis of H_2_O_2_ (Figure 3).

This result indicates that the SC-CO_2_-MD extract contributes to the protection of certain structural forms of DNA against OH∙ radicals generated by the UV photolysis of H_2_O_2_. Although it was observed that the supercoiled form (Form I) could not be fully protected, the effect on the open circular (Form II) and linear (Form III) forms indicates that the extract exhibits partial DNA-protective activity. In conclusion, the garlic extract obtained via SC-CO_2-_MD has demonstrated a certain level of protective effect in maintaining the structural integrity of plasmid DNA and has revealed potential bioprotective properties against hydroxyl radical-induced oxidative DNA damage (Figure 3).

## 4. Conclusions

This study presents a novel and environmentally friendly strategy for producing high-purity allicin, the major bioactive compound of garlic, while maintaining its chemical stability. The combined use of SC-CO_2_ extraction and molecular distillation provides an effective system that balances extraction efficiency with product purity. Optimization results indicated that the most suitable SC-CO_2_ conditions were 150 bar pressure, 65% (*v*/*v*) ethanol concentration, and a flow rate of 0.5 mL/min, yielding an allicin concentration of 5745.55 mg/L. Further purification by molecular distillation significantly increased allicin purity to 67.10%, with optimal parameters of an evaporator temperature of 79 °C and a feed rate of 2.025 mL/min. While the initial SC-CO_2_ process resulted in an allicin purity of 45.77%, the subsequent MD step markedly improved this value. Bioactivity assessments demonstrated that purified allicin exhibits strong antimicrobial activity against both Gram-positive and Gram-negative bacteria, as well as superior antifungal activity against *Aspergillus* and *Penicillium* species compared with Nystatin. It has been determined that allicin undergoes complete structural degradation in the intestinal system under duodenal conditions and becomes completely undetectable in intestinal contents.

In vitro gastrointestinal digestion results demonstrated that garlic extracts obtained by SC-CO_2_ extraction exhibited the highest antioxidant capacity and total phenolic content during the gastric phase, while the bioaccessibility and stability of allicin were markedly reduced throughout digestion, particularly under gastric and intestinal conditions, highlighting the critical influence of gastrointestinal pH on the fate of this bioactive organosulfur compound.

The allicin-rich garlic extracts obtained by SC-CO_2_ extraction and subsequent molecular distillation demonstrated partial protection against oxidative DNA damage by preserving specific plasmid DNA conformations, suggesting their potential as natural bioprotective agents against reactive oxygen species-induced genetic damage.

These findings provide an important scientific basis for the potential utilization of allicin-rich extracts in food-related applications and offer valuable insights for future research. In particular, the development of advanced delivery systems, such as nanoencapsulation or liposomal carriers, will be essential to overcome challenges related to allicin’s instability and volatility, thereby improving its shelf life and enabling controlled release. Finally, evaluating the economic viability and scalability of this green extraction and purification approach will play a key role in transforming these findings into commercially viable, value-added products.

## Figures and Tables

**Table 1:** (**a**) Process variables used in Box–Behnken design for SC-CO_2_ extraction. (**b**) Experimental design applied for the SC-CO_2_ extraction method. (**c**) Process variables used in Box–Behnken design for MD. (**d**) Experimental design for the molecular distillation method.

(**a**)										
						**Variable Level Codes**				
					**Code**	**−1**	**0**			**+1**
Pressure (bar)					X_1_	150	225			300
Co-solvent concentration (%)					X_2_	50	65			80
Flow rate (mL/min)					X_3_	0.50	1.75			3
(**b**)										
**Code Values**						**True Values**				
**Run**		**X_1_**	**X_2_**		**X_3_**	**Pressure (Bar)**	**Co-Solvent Concentration (%)**			**Flow Rate (mL/min)**
1		−1	0		−1	150	65			0.5
2		1	0		−1	300	65			0.5
3		0	0		0	225	65			1.75
4		1	−1		0	300	50			1.75
5		−1	0		1	150	65			3
6		−1	1		0	150	80			1.75
7		0	0		0	225	65			1.75
8		1	1		0	300	80			1.75
9		0	−1		1	225	50			3
10		−1	−1		0	150	50			1.75
11		0	0		0	225	65			1.75
12		0	0		0	225	65			1.75
13		0	1		−1	225	80			0.5
14		0	−1		−1	225	50			0.5
15		0	1		1	225	80			3
16		0	0		0	225	65			1.75
17		1	0		1	300	65			3
(**c**)										
							**Variable Level Codes**			
						**Code**	**−1**		**0**	**1**
Wiper Speed (rpm)						X_1_	100		160	220
Feed Rate (mL/min)						X_2_	2.00		3.00	4.00
Pressure (mbar)						X_3_	70.00		160	250
Evaporator Unit Temperature (°C)						X_4_	30.00		55.00	80.00
Condenser Temperature (°C)						X_5_	10.00		15.00	20.00
(**d**)										
**Code Values**						**True Values**				
**Run**	**X_1_**	**X_2_**	**X_3_**	**X_4_**	**X_5_**	**Wiper Speed (rpm)**	**Feed Rate (mL/min)**	**Pressure (mbar)**	**Evaporator Unit** **Temperature (°C)**	**Condenser** **Temperature (°C)**
1	0	0	1	0	1	160	3	250	55	20
2	1	−1	0	0	0	220	2	160	55	15
3	0	0	−1	0	1	160	3	70	55	20
4	0	1	0	0	1	160	4	160	55	20
5	0	1	0	0	0	160	4	160	30	15
6	1	0	0	−1	0	220	3	160	30	15
7	1	0	0	1	0	220	3	160	80	15
8	0	0	0	−1	1	160	3	160	30	20
9	0	0	−1	0	−1	160	3	70	55	10
10	0	0	0	−1	−1	160	3	160	30	10
11	0	0	0	0	0	160	3	160	55	15
12	0	0	1	1	0	160	3	250	80	15
13	0	1	0	0	−1	160	4	160	55	10
14	−1	−1	0	0	0	100	2	160	55	15
15	−1	0	0	0	1	100	3	160	55	20
16	0	1	1	0	0	160	4	250	55	15
17	0	−1	0	1	0	160	2	160	80	15
18	0	0	1	0	−1	160	3	250	55	10
19	0	−1	−1	0	0	160	2	70	55	15
20	1	0	1	0	0	220	3	250	55	15
21	0	0	−1	−1	0	160	3	70	30	15
22	0	0	0	1	1	160	3	160	80	20
23	1	0	0	0	−1	220	3	160	55	10
24	−1	0	0	1	0	100	3	160	80	15
25	0	0	0	0	0	160	3	160	55	15
26	0	0	0	0	0	160	3	160	55	15
27	0	0	0	1	−1	160	3	160	80	10
28	−1	0	−1	0	0	100	3	70	55	15
29	1	0	0	0	1	220	3	160	55	20
30	0	−1	0	0	−1	160	2	160	55	10
31	0	0	0	0	0	160	3	160	55	15
32	0	−1	0	0	1	160	2	160	55	20
33	0	−1	0	−1	0	160	2	160	30	15
34	1	1	0	0	0	220	4	160	55	15
35	0	1	−1	0	0	160	4	70	55	15
36	−1	1	0	0	0	100	4	160	55	15
37	0	0	1	−1	0	160	3	250	30	15
38	−1	0	1	0	0	100	3	250	55	15
39	−1	0	0	−1	0	100	3	160	30	15
40	1	0	−1	0	0	220	3	70	55	15
41	0	−1	1	0	0	160	2	250	55	15
42	−1	0	0	0	−1	100	3	160	55	10
43	0	0	0	0	0	160	3	160	55	15
44	0	1	0	1	0	160	4	160	80	15
45	0	0	−1	1	0	160	3	70	80	15

**Table 2:** Physicochemical analysis results.

	SC-CO_2_	SC-CO_2_-MD
pH	6.53 ± 0.01	7.67 ± 0.15
Color Parameters		
L*	94.60 ± 0.01	95.50 ± 0.02
a*	−3.65 ± 0.01	−2.57 ± 0.01
b*	15.43 ± 0.01	5.15 ± 0.01
*C*	15.86 ± 0.01	5.75 ± 0.01
h*	103.32 ± 0.02	116.56 ± 0.03
Total Phenolic Content (TPC) (TFM) ^1^	595.99 ± 2.81	117.90 ± 1.86
DPPH ^2^	621.85 ± 5.72	32.15 ± 0.01
ABTS ^2^	2008.50 ± 5.34	270.01 ± 2.29
CUPRAC ^3^	5.27 ± 0.33	0.12 ± 0.01
FRAP ^4^	181.81 ± 1.65	102.36 ± 0.05

^1^ mg GAE/L, ^2^ μmol Trolox/L, ^3^ mM Trolox/L, ^4^ mg Trolox/L.

**Table 3:** Antibacterial and antifungal activities of SC-CO_2_ and SC-CO_2_-MD samples against selected microorganisms (inhibition zone diameter, mm).

	Positive Control	SC-CO_2_	SC-CO_2_-MD
*E. coli*	24.83 ± 0.07 *	35.67 ± 0.01	27.51 ± 0.10
*S. aureus*	21.67 ± 0.01 *	44.17 ± 0.27	37.53 ± 0.18
*B. subtilis*	23.33 ± 0.10 *	32.33 ± 0.57	31.56 ± 0.05
*P. aeruginosa*	15.00 ± 0.01 *	28.33 ± 0.55	12.55 ± 0.06
*K. pneumoniae*	24.33 ± 0.10 *	28.83 ± 0.21	20.73 ± 0.10
*A. fumigatus*	20.83 ± 0.05 **	29.38 ± 0.01	27.51 ± 0.10
*P. chrysogenum*	20.26 ± 0.04 **	29.12 ± 0.12	17.53 ± 0.18
*C. cladosporioides*	20.07 ± 0.03 **	18.74 ± 0.19	11.56 ± 0.05
*Alternaria* species	22.39 ± 0.01 **	29.38 ± 0.01	12.55 ± 0.06

* Gentamicin, ** Nystatin.

**Table 4:** Bioactivity analysis results of oral, gastric, and intestinal phases following in vitro digestion.

		SC-CO_2_			SC-CO_2_-MD		
		Oral	Gastric	Intestinal	Oral	Gastric	Intestinal
TPC ^1^	Younger adult	516.79 ± 0.31 ^b^	587.83 ± 2.82 ^a^	183.55 ± 0.77 ^c^	194.18 ± 0.34 ^b^	273.58 ± 0.23 ^a^	75.15 ± 0.52 ^c^
	Older adult	515.62 ± 1.80 ^b^	582.08 ± 6.20 ^a^	179.49 ± 9.97 ^c^	194.14 ± 3.11 ^b^	250.82 ± 0.23 ^a^	69.75 ± 0.52 ^c^
DPPH ^2^	Younger adult	614.10 ± 12.35	902.00 ± 22.02 ^a^	85.80 ± 1.11 ^c^	251.43 ± 3.02 ^b^	728.38 ± 6.98 ^a^	32.00 ± 1.98 ^c^
	Older adult	588.38 ± 5.43 ^b^	869.98 ± 2.86 ^a^	95.83 ± 2.23 ^c^	267.81 ± 5.64 ^b^	689.52 ± 1.32 ^a^	25.14 ± 1.98 ^c^
ABTS ^2^	Younger adult	1964.12 ± 26.89 ^b^	2366.45 ± 21.61 ^a^	174.72 ± 4.68 ^c^	674.93 ± 1.85 ^b^	1337.60 ± 7.33 ^a^	92.40 ± 1.70 ^c^
	Older adult	1978.70 ± 12.43 ^b^	2583.60 ± 10.00 ^a^	161.46 ± 3.90 ^c^	627.47 ± 3.23 ^b^	1277.87 ± 9.38 ^a^	75.20 ± 2.77 ^c^
CUPRAC ^3^	Younger adult	1.83 ± 0.01 ^b^	2.62 ± 0.01 ^a^	0.27 ± 0.01 ^c^	0.75 ± 0.03 ^b^	1.90 ± 0.07 ^a^	0.21 ± 0.00 ^c^
	Older adult	1.84 ± 0.02 ^b^	2.28 ± 0.04 ^a^	0.15 ± 0.03 ^c^	0.67 ± 0.01 ^b^	1.68 ± 0.01 ^a^	0.06 ± 0.04 ^c^
FRAP ^4^	Younger adult	171.50 ± 2.13 ^b^	188.73 ± 1.72 ^a^	80.59 ± 0.59 ^c^	108.24 ± 0.39 ^b^	115.96 ± 0.59 ^a^	56.20 ± 0.05 ^c^
	Older adult	169.47 ± 0.20 ^b^	186.67 ± 0.56 ^a^	65.90 ± 0.24 ^c^	92.07 ± 0.07 ^b^	94.28 ± 0.48 ^a^	47.10 ± 0.54 ^c^
Allicin Content	Younger adult	5008.31 ± 22.60 ^a^	3593.29 ± 9.47 ^b^	172.71 ± 3.09 ^c^	927,71 ± 4,79 ^b^	762.56 ± 18.78 ^b^	ND
	Older adult	4917.48 ± 22.30 ^a^	3749.76 ± 12.43 ^b^	180.00 ± 0.02 ^c^	923,80 ± 9,17 ^b^	719.11 ± 9.36 ^b^	ND
*E. coli*	Younger adult	26.50 ± 0.05 ^a^	26.00 ± 0.01 ^a^	NI	NI	NI	NI
	Older adult	25.83 ± 0.01 ^a^	21.50 ± 0.57 ^b^	NI	NI	NI	NI
*S. aureus*	Younger adult	36.33 ± 0.50 ^a^	31.33 ± 0.01 ^b^	NI	NI	NI	NI
	Older adult	34.67 ± 0.10 ^a^	31.33 ± 0.01 ^b^	NI	NI	NI	NI
*B. subtilis*	Younger adult	17.50 ± 0.10 ^a^	14.50 ± 0.50 ^b^	NI	NI	NI	NI
	Older adult	18.50 ± 0.50 ^a^	11.50 ± 0.57 ^b^	NI	NI	NI	NI
*P. aeruginosa*	Younger adult	23.00 ± 0.01 ^b^	24.17 ± 0.10 ^a^	NI	NI	NI	NI
	Older adult	23.50 ± 0.50 ^b^	26.00 ± 0.50 ^a^	NI	NI	NI	NI
*K. pneumoniae*	Younger adult	13.67 ± 0.10 ^a^	13.83 ± 0.10 ^a^	NI	NI	NI	NI
	Older adult	14.17 ± 0.10 ^a^	11.67 ± 0.10 ^b^	NI	NI	NI	NI

^a–c^ Different superscript letters within the same column indicate statistically significant differences among the oral, gastric, and intestinal phases for each sample (*p* < 0.05). ^1^ mg GAE/L, ^2^ μmol Trolox/L, ^3^ mM Trolox/L, ^4^ mg Trolox/L. ND: Not detected. NI: No inhibition observed. No antifungal activity was observed.

## Data Availability

The original contributions presented in the study are included in the article and Supplementary Material, further inquiries can be directed to the corresponding author.

## Supplementary Materials

The following supporting information can be downloaded at https://www.mdpi.com/article/10.3390/foods15122174/s1: Table S1: Independent variables of the SC-CO_2_ extraction method (pressure (bar), co-solvent concentration (%), flow rate (mL/min), and corresponding responses: allicin content, total sulphur-containing compound content (TSC), total phenolic content (TPC), antioxidant capacity (DPPH and ABTS) results, Table S2: ANOVA table for the reduced model showing the individual effects of the linear and interaction terms of all factors (pressure, co-solvent concentration, and flow rate) on the responses (DPPH), Table S3: Terms used for testing model adequacy in the reduced model (DPPH), Table S4: ANOVA table for the reduced model showing the individual effects of the linear and interaction terms of all factors (pressure, co-solvent concentration, and flow rate) on the responses (ABTS), Table S5: Terms used for testing model adequacy in the reduced model (ABTS), Table S6: ANOVA table for the reduced model showing the individual effects of the linear and interaction terms of all factors (pressure, co-solvent concentration, and flow rate) on the responses (TPC), Table S7: Terms used for testing model adequacy in the reduced model (TPC), Table S8: ANOVA table for the reduced model showing the individual effects of the linear and interaction terms of all factors (pressure, co-solvent concentration, and flow rate) on the responses (Allicin), Table S9: Terms used for testing model adequacy in the reduced model (Allicin), Table S10: ANOVA table for the reduced model showing the individual effects of the linear and interaction terms of all factors (pressure, co-solvent concentration, and flow rate) on the responses (TSC), Table S11: Terms used for testing model adequacy in the reduced model (TSC), Table S12: Independent variables and experimental conditions of the molecular distillation process (wiper speed, feed rate, pressure, evaporator temperature, and condenser temperature), Table S13: ANOVA Table for the reduced model showing the individual effects of linear and interaction terms of all factors (wiper speed, feed rate, pressure, evaporator temperature, and condenser temperature) on the responses, Table S14: Terms used for testing model adequacy in the reduced model (Allicin purity).

## Abbreviations

The following abbreviations are used in this manuscript:

SC-CO_2_	Supercritical-Carbon Dioxide
MD	Molecular distillation
DPPH	2-diphenyl-1-picrylhydrazyl
ABTS	2,2′-azino-bis(3-ethylbenzothiazoline-6-sulfonic acid)
TPC	Total phenolic content
TSC	Total sulfur compounds
GAE	Gallic acid equivalents
