# Supplementary material for: Enhancing Allicin Purity and Gastrointestinal Bioactivity Profile of Garlic Extracts Through Optimized Supercritical-CO2 Extraction and Molecular Distillation Processes
Source: Foods. 2026 Jun 16;15(12):2174. doi: 10.3390/foods15122174 (PMC13297873; doi:10.3390/foods15122174)
Supplement: Supplementary file 1 [file foods-15-02174-s001.zip › foods-4338942-supplementary.pdf]

SUPPLEMENTARY TABLES

Table S1. Independent variables of the SC-CO_2 extraction method (pressure (bar), co-solvent concentration (%), flow rate (mL/min), and corresponding responses: allicin content, total sulphur-containing compound content (TSC), total phenolic content (TPC), antioxidant capacity (DPPH and ABTS) results.

Table S2. ANOVA table for the reduced model showing the individual effects of the linear and interaction terms of all factors (pressure, co-solvent concentration, and flow rate) on the responses (DPPH).

Table S3. Terms used for testing model adequacy in the reduced model (DPPH).

Table S4. ANOVA table for the reduced model showing the individual effects of the linear and interaction terms of all factors (pressure, co-solvent concentration, and flow rate) on the responses (ABTS).

Table S5. Terms used for testing model adequacy in the reduced model (ABTS).

Table S6. ANOVA table for the reduced model showing the individual effects of the linear and interaction terms of all factors (pressure, co-solvent concentration, and flow rate) on the responses (TPC).

Table S7. Terms used for testing model adequacy in the reduced model (TPC).

Table S8. ANOVA table for the reduced model showing the individual effects of the linear and interaction terms of all factors (pressure, co-solvent concentration, and flow rate) on the responses (Allicin).

Table S9. Terms used for testing model adequacy in the reduced model (Allicin).

Table S10. ANOVA table for the reduced model showing the individual effects of the linear and interaction terms of all factors (pressure, co-solvent concentration, and flow rate) on the responses (TSC).

Table S11. Terms used for testing model adequacy in the reduced model (TSC).

Table S12. Independent variables and experimental conditions of the molecular distillation process (wiper speed, feed rate, pressure, evaporator temperature, and condenser temperature).

Table S13. ANOVA Table for the reduced model showing the individual effects of linear and interaction terms of all factors (wiper speed, feed rate, pressure, evaporator temperature, and condenser temperature) on the responses.

Table S14. Terms used for testing model adequacy in the reduced model (Allicin purity).

Table S1. Independent variables of the SC-CO_2 extraction method (pressure (bar), co-solvent concentration (%), flow rate (mL/min), and corresponding responses: allicin content, total sulphur-containing compound content (TSC), total phenolic content (TPC), antioxidant capacity (DPPH and ABTS) results.

**Run**	**Pressure (bar)**	**Co-Solvent Concentration (%)**	**Flow rate (mL/min)**	**DPPH (µmol Trolox /L)**	**ABTS (µmol Trolox /L)**	**TPC (mg GAE /L)**	**Allicin (mg/L)**	**TSC (mg/L)**
**1**	150	65	0.5	605.28±1.61	2155.42±2.37	555.61±1.13	5745.55±2.04	16395.20±7.41
**2**	300	65	0.5	349.38±0.39	845.55±1.91	392.12±0.61	2674.35±2.69	8833.65±3.17
**3**	225	65	1.75	335.95±0.66	593.05±1.32	306.64±0.10	1903.21±1.35	5869.72±2.52
**4**	300	50	1.75	248.95±0.10	606.94±1.02	317.94±0.77	2145.53±1.13	6140.61±3.13
**5**	150	65	3	229.95±0.43	637.22±0.75	172.46±0.19	1204.25±1.13	3485.88±3.14
**6**	150	80	1.75	327.95±0.24	703.33±1.14	266.64±0.07	2271.69±1.07	6632.64±2.87
**7**	225	65	1.75	269.62±0.50	596.38±0.72	255.34±0.26	1404.73±2.30	4768.72±1.05
**8**	300	80	1.75	337.00±0.34	704.72±1.14	278.69±0.16	2003.61±1.62	6746.44±3.62
**9**	225	50	3	233.57±0.79	705.27±1.73	212.87±0.38	1387.73±1.32	4040.12±3.32
**10**	150	50	1.75	245.47±0.90	766.38±1.32	290.27±0.23	1714.77±1.22	4727.26±1.22
**11**	225	65	1.75	313.19±0.40	769.16±1.27	307.12±0.42	1827.19±1.22	4805.23±1.21
**12**	225	65	1.75	321.47±0.47	757.77±1.65	319.52±0.98	2242.99±2.32	6802.66±1.90
**13**	225	80	0.5	544.57±1.41	1182.50±1.67	490.82±0.45	4659.98±2.56	12126.70±3.62
**14**	225	50	0.5	551.57±1.25	1441.30±1.92	529.58±0.13	2498.29±1.62	8012.34±3.42
**15**	225	80	3	267.90±0.76	531.94±0.65	186.84±0.97	1357.40±1.31	3995.15±3.07
**16**	225	65	1.75	351.00±0.52	733.61±0.28	247.46±0.33	2264.82±1.61	6773.65±3.31
**17**	300	65	3	287.76±0.20	827.50±0.73	249.52±0.47	1343.1±1.07	4181.52±4.21

Response values are given as mean ± standard deviation.

Table S2. ANOVA table for the reduced model showing the individual effects of the linear and interaction terms of all factors (pressure, co-solvent concentration, and flow rate) on the responses (DPPH).

Source	Sum of Squares	*df*	Mean Square	*F* Value	*p* Value
Model	1.878E+05	4	46947.28	23.39	< 0.0001
A	4304.59	1	4304.59	2.14	0.1688
C	1.330E+05	1	1.330E+05	66.28	< 0.0001
AC	24604.16	1	24604.16	12.26	0.0044
C2	25850.62	1	25850.62	12.88	0.0037
Residual	24085.66	12	2007.14		
Lack of fit	20298.74	8	2537.34	2.68	0.1783
Pure error	3786.91	4	946.73		
Cor total	2.119E+05	16			

Table S3. Terms used for testing model adequacy in the reduced model (DPPH).

Standard Deviation	44.80
Mean	342.39
R-Squared	0.8863
Adj R-Squared	0.8484
Pred R-Squared	0.6787
Adeq Precision	17.0706
C.V. %	13.08
PRESS	68070.09

Table S4. ANOVA table for the reduced model showing the individual effects of the linear and interaction terms of all factors (pressure, co-solvent concentration, and flow rate) on the responses (ABTS).

Source	Sum of Squares	*df*	Mean Square	*F* Value	*p* Value
Model	2,349E+06	4	5,872E+05	28,15	<0.0001
A	2,040E+05	1	2,040E+05	9,78	0.0087
C	1,068E+06	1	1,068E+06	51,19	<0.0001
AC	5,626E+05	1	5,626E+05	26,97	0.0002
C^2	5,143E+05	1	5,143E+05	24,65	0.0003
Residual	2,503E+05	12	20862,13		
Lack of fit	2,194E+05	8	27427,64	3,55	0.1183
Pure error	30924,38	4	7731,10		
Cor total	2,599E+06	16			

Table S5. Terms used for testing model adequacy in the reduced model (ABTS).

Standard Deviation	144.44
Mean	856.36
R-Squared	0.9037
Adj R-Squared	0.8716
Pred R-Squared	0.6682
Adeq Precision	18.9041

C.V. %	16.87
PRESS	8.625E+05

Table S6. ANOVA table for the reduced model showing the individual effects of the linear and interaction terms of all factors (pressure, co-solvent concentration, and flow rate) on the responses (TPC).

Source	Sum of Squares	df	Mean Square	F Value	*p* Value
Model	1.948E+05	4	48696.17	58.50	<0.0001
A	272.76	1	272.76	0.3276	0.5776
C	1.643E+05	1	1.643E+05	197.35	<0.0001
AC	14465.83	1	14465.83	17.38	0.0013
C^2	15755.99	1	15755.99	18.93	0.0009
Residual	9989.71	12	832.48		
Lack of fit	5576.38	8	697.05	0.6318	0.7313
Pure error	4413.33	4	1103.33		
Cor total	2.048E+05	16			

Table S7. Terms used for testing model adequacy in the reduced model (TPC).

Standard Deviation	28.85
Mean	316.44
R-Squared	0.9512
Adj R-Squared	0.9350
Pred R-Squared	0.8802
Adeq Precision	26.0030
C.V. %	9.12
PRESS	24524.34

Table S8. ANOVA table for the reduced model showing the individual effects of the linear and interaction terms of all factors (pressure, co-solvent concentration, and flow rate) on the responses (Allicin).

Source	Sum of Squares	*df*	Mean Square	*F* Value	*p* Value
Model	1.676E+07	3	5.586E+06	11.48	0.0006
A	9.589E+05	1	9.589E+05	1.97	0.1839
C	1.322E+07	1	1.322E+07	27.17	0.0002
AC	2.576E+06	1	2.576E+06	5.29	0.0386
Residual	6.328E+06	13	4.868E+05		
Lack of fit	5.831E+06	9	6.479E+05	5.21	0.0634
Pure error	4.973E+05	4	1.243E+05		
Cor total	2.309E+07	16			

Table S9. Terms used for testing model adequacy in the reduced model (Allicin).

Standard Deviation	697.71
Mean	2273.48
R-Squared	0.7259
Adj R-Squared	0.6626
Pred R-Squared	0.3527
Adeq Precision	12.3403
C.V. %	30.69
PRESS	1.495E+07

Table S10. ANOVA table for the reduced model showing the individual effects of the linear and interaction terms of all factors (pressure, co-solvent concentration, and flow rate) on the responses (TSC).

**Source**	**Sum of Squares**	***df***	**Mean Square**	***F* Value**	***p* Value**
Model	1.431E+08	4	3.577E+07	14.34	0.0002
A	3.563E+06	1	3.563E+06	1.43	0.2552
C	1.100E+08	1	1.100E+08	44.09	<0.0001
AC	1.705E+07	1	1.705E+07	6.83	0.0226
C^2	1.246E+07	1	1.246E+07	4.99	0.0452
Residual	2.994E+07	12	2.495E+06		
Lack of fit	2.593E+07	8	3.241E+06	3.23	0.1361
Pure error	4.011E+06	4	1.003E+06		
Cor total	1.730E+08	16			

Table S11. Terms used for testing model adequacy in the reduced model (TSC).

Standard Deviation	1579.51
Mean	6725.73
R-Squared	0.8270
Adj R-Squared	0.7693
Pred R-Squared	0.4708
Adeq Precision	13.4774
C.V. %	23.48
PRESS	9.156E+07

Table S12. Independent variables and experimental conditions of the molecular distillation process (wiper speed, feed rate, pressure, evaporator temperature, and condenser temperature).

Run	Wiper Speed (rpm)	Feed Rate (mL/min)	Pressure (mbar)	Evaporator Temperature (°C)	Condenser Temperature (°C)	Allicin Purity (%)
1	160	3	250	55	20	54.75±0.09
2	220	2	160	55	15	61.82±0.03
3	160	3	70	55	20	62.45±0.05
4	160	4	160	55	20	60.82±0.37
5	160	4	160	30	15	49.40±0.32
6	220	3	160	30	15	45.59±0.03
7	220	3	160	80	15	61.71±0.28
8	160	3	160	30	20	51.73±0.58
9	160	3	70	55	10	65.99±0.05
10	160	3	160	30	10	53.41±0.27
11	160	3	160	55	15	60.28±0.52
12	160	3	250	80	15	63.21±0.53
13	160	4	160	55	10	65.42±0.50
14	100	2	160	55	15	63.34±0.15
15	100	3	160	55	20	65.04±0.11
16	160	4	250	55	15	50.43±0.07
17	160	2	160	80	15	69.19±0.09
18	160	3	250	55	10	54.36±0.10
19	160	2	70	55	15	64.50±0.18
20	220	3	250	55	15	49.81±0.03
21	160	3	70	30	15	66.49±0.10
22	160	3	160	80	20	68.32±0.03
23	220	3	160	55	10	61.66±0.08
24	100	3	160	80	15	66.40±0.09
25	160	3	160	55	15	61.87±0.35
26	160	3	160	55	15	62.05±0.63
27	160	3	160	80	10	65.24±0.22
28	100	3	70	55	15	61.57±0.02
29	220	3	160	55	20	62.64±0.22
30	160	2	160	55	10	66.01±0.17
31	160	3	160	55	15	59.45±0.06
32	160	2	160	55	20	63.48±0.45
33	160	2	160	30	15	58.21±0.10
34	220	4	160	55	15	59.42±0.62
35	160	4	70	55	15	63.50±0.42
36	100	4	160	55	15	59.21±0.24
37	160	3	250	30	15	49.50±0.34
38	100	3	250	55	15	54.32±0.14
39	100	3	160	30	15	51.16±0.51
40	220	3	70	55	15	63.87±0.06
41	160	2	250	55	15	62.90±0.28
42	100	3	160	55	10	64.28±0.31
43	160	3	160	55	15	61.19±0.14
44	160	4	160	80	15	66.42±0.07
45	160	3	70	80	15	62.59±0.12

Response values are expressed as mean ± standard deviation.

Table S13. ANOVA Table for the reduced model showing the individual effects of linear and interaction terms of all factors (wiper speed, feed rate, pressure, evaporator temperature, and condenser temperature) on the responses.

Source	Sum of Squares	*df*	Mean Square	*F* Value	*p* Value
Model	931.42	11	84.67	18.65	<0.0001
A-A	8.95	1	8.95	1.97	0.1706
B-B	49.21	1	49.21	10.84	0.0025
C-C	385.00	1	385.00	84.81	<0.0001
D-D	438.53	1	438.53	96.61	<0.0001
E-E	3.19	1	3.19	0.7027	0.4085
AD	32.86	1	32.86	7.24	0.0115
CD	21.66	1	21.66	4.77	0.0369
D^2	141.83	1	141.83	31.24	<0.0001
Residual	48.48	1	48.48	10.68	0.0027
Lack of fit	46.90	1	46.90	10.33	0.0031
Pure error	22.20	1	22.20	4.89	0.0348
Cor total	136.18	30	4.54		

Table S14. Terms used for testing model adequacy in the reduced model (Allicin purity).

Standard Deviation	2.13
Mean	60.73
R-Squared	0.8724
Adj R-Squared	0.8257
Pred R-Squared	0.6566
Adeq Precision	16.5620
C.V. %	3.51
PRESS	366.63
